# The Role of Macrophages in Cancer: From Basic Research to Clinical Applications

**DOI:** 10.1002/mco2.70547

**Published:** 2025-12-19

**Authors:** Zhimei Liu, Yan Li, Jingchao Cao, Yefeng Qiu, Kun Yu, Shoulong Deng

**Affiliations:** ^1^ Beijing Key Laboratory for Animal Genetic Improvement, National Engineering Laboratory for Animal Breeding, Key Laboratory of Animal Genetics and Breeding of the Ministry of Agriculture, College of Animal Science and Technology, National Research Facility for Phenotypic and Genotypic Analysis of Model Animals (Beijing) China Agricultural University Beijing China; ^2^ Academy of Military Medical Sciences Academy of Military Sciences Beijing China; ^3^ National Center of Technology Innovation for Animal Model, National Human Diseases Animal Model Resource Center, National Health Commission of China (NHC) Key Laboratory of Comparative Medicine, Institute of Laboratory Animal Sciences Chinese Academy of Medical Sciences and Comparative Medicine Center, Peking Union Medical College Beijing China

**Keywords:** CAR‐M, cell therapy, macrophages, tumor‐associated macrophages, tumor microenvironment

## Abstract

Macrophages are innate immune cells that extensively infiltrate and play a key role in the tumor microenvironment (TME). Tumor cell–secreted factors recruit monocytes into the TME, where they differentiate into tumor‐associated macrophages (TAMs), which can polarize into distinct phenotypes: M1 and M2. M1 TAMs promote antitumor immunity through cytokine secretion and antigen presentation, whereas M2 TAMs support tumor progression by facilitating angiogenesis, invasion, and immune escape. Despite these dual roles, the specific mechanisms governing macrophage plasticity and polarization remain insufficiently understood. This review comprehensively summarizes the origin, polarization, and functional diversity of macrophages in the TME, with emphasis on pathways that regulate TAM‐mediated immune responses. Furthermore, this article examines current TAM‐targeted therapeutic strategies, including recruitment inhibition, phenotypic reprogramming, and the development of chimeric antigen receptor macrophages (CAR‐Ms), as well as macrophage‐based drug delivery and exosome therapy. By integrating recent advances in cell engineering and immunometabolism, this review highlights the translational potential of TAM‐targeted therapies and their value in reshaping the immunosuppressive TME to enhance cancer immunotherapy.

## Graphical abstract

Monocytes infiltrate the tumor microenvironment (TME) and differentiate into macrophages, which polarize into proinflammatory, antitumor M1 cells or immunosuppressive, protumor M2 cells. Within the TME, M2‐type tumor‐associated macrophages (TAMs) predominate and drive tumor growth, angiogenesis, and metastasis. Therapeutic strategies therefore focus on depleting or reprogramming TAMs (including chimeric antigen receptor macrophages [CAR‐Ms] and macrophage/exosome‐based drug delivery), while TAM‐related biomarkers such as the CD68/CD163 ratio, single‐cell RNA sequencing (scRNA‐seq), spatial transcriptomics, and cytometry by time‐of‐flight offer diagnostic and prognostic value.

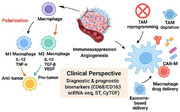

## 1 Introduction

Macrophages participate in essential immune processes in the body and play key roles in human health and disease. As innate immune cells, they contribute to the regulation of various immune‐related disorders in response to microbial invasion, tissue damage, and metabolic disturbances. Macrophages are present in nearly all tissues and exhibit dynamic responses; they adapt to diverse stimuli through the generation of distinct functional subsets. These subsets can be broadly classified into the classic M1 type, the alternatively activated M2 type, and the M2‐like type [1]. Tissue‐resident macrophages (TRMs) and monocyte‐derived macrophages both participate in innate immune responses, providing protection to the host. They also cooperate with other innate immune cells, such as neutrophils and innate lymphocytes (e.g., natural killer [NK] cells). Due to their high plasticity, TRM populations respond to local signals while maintaining core phagocytic function and acquire specific functions required to sustain tissue homeostasis [2, 3]. Macrophage plasticity is a key factor in chronic inflammation and is closely associated with multiple human diseases, especially cancer. Distinct macrophage subsets play specific roles in the tumor microenvironment (TME), contributing to matrix remodeling, angiogenesis, and the promotion of tumor invasion and metastasis. Macrophage‐based cancer treatment strategies are increasingly recognized as promising therapeutic approaches. Macrophages can serve as carriers for liposomes or nanoparticles loaded with chemotherapeutic agents such as doxorubicin. Exogenous factors can reprogram tumor‐associated macrophages (TAMs) from the M2 phenotype to a proinflammatory M1 phenotype. Additionally, TAMs can secrete exosomes containing programmed cell death ligand 1 (PD‐L1); methods that target TAMs or their exosomes can inhibit tumor progression [4].

As research advances, the importance of TAMs has become increasingly evident. They infiltrate tumor tissue within the TME and influence the trajectory of tumor progression. Therefore, this review summarizes recent progress in understanding TAM recruitment and repolarization and discusses emerging therapeutic strategies targeting macrophages. Current approaches primarily focus on eliminating immunosuppressive TAMs or enhancing macrophage phagocytic activity. With the development of biotechnology and gene‐editing techniques, methods such as chimeric antigen receptor macrophage (CAR‐M) engineering are rapidly progressing and currently undergoing clinical evaluation.

Given the multifaceted roles macrophages play in cancer progression, we aim to provide a comprehensive overview of the biological characteristics and therapeutic potential of TAMs. First, we summarize recent advances in research on the origin, recruitment, and phenotypic polarization of macrophages in the TME, highlighting the molecular and metabolic pathways that shape their diverse functions. We then discuss current and emerging therapeutic strategies targeting TAMs, including suppression of immunosuppressive M2‐like macrophage populations, reprogramming of macrophage phenotypes to a proinflammatory state, and the development of CAR‐Ms. Furthermore, we examine macrophage‐derived exosomes and nanocarrier systems as innovative tools for drug delivery and immunomodulation. By integrating these perspectives, we establish a conceptual framework that bridges basic macrophage biology and translational oncology, offering new insights into macrophage‐centered cancer immunotherapy.

## 2 Macrophages

Macrophages are among the most functionally diverse immune cell populations in the body; they play crucial roles in tissue development, host defense, and homeostasis. Single‐cell technologies have revealed previously unrecognized complexity within this lineage, leading to increasing attention toward the origin and functional diversity of macrophages [5]. This section traces the developmental origins of macrophages—from embryonic yolk sac progenitors to hematopoietic stem cell‐derived monocytes—and examines how these distinct lineages give rise to tissue‐resident and circulating macrophages. It then summarizes various macrophage subsets, including classic M1/M2 polarization states, immunomodulatory macrophages, and specialized CD169+ and T‐cell receptor (TCR)+ populations, each with a distinct role in immune homeostasis. Finally, this section focuses on TAMs, whose functional plasticity connects macrophage biology to cancer progression and therapeutic intervention.

### 2.1 Macrophage Origin and Development

Macrophages arise from multiple sources. Initially, circulating monocytes were considered the primary origin of TRMs [6]. These monocytes develop from hematopoietic stem cells; however, during embryonic development, macrophages also emerge in the fetal liver, yolk sac, and regions near the dorsal aorta. Consequently, many adult macrophages develop independently of monocytes [7]. With advances in single‐cell sequencing, the origins of macrophages in human embryos have become clearer; their distinct functions correspond to their individual developmental pathways. Most TRMs primarily originate from yolk sac–derived myeloid‐biased progenitors or from hematopoietic stem cells and their progenitors [8, 9]. Single‐cell RNA sequencing (scRNA‐seq) has provided further clarity regarding macrophage developmental origins. For example, Eraslan et al. identified a conserved macrophage developmental pathway in which mononuclear precursors differentiate into macrophages with high major histocompatibility complex (MHC)‐II expression and immune functions in tissues, as well as macrophages characterized by high lymphatic vessel endothelial hyaluronan receptor 1 expression that support vasculature and limit immune cell infiltration [10]. Additionally, a comprehensive reference database of immune cell types has revealed that macrophages expressing erythrophagocytosis‐related genes are widely distributed in the spleen, liver, bone marrow, and lymph nodes [11]. In the mouse yolk sac, two main differentiation pathways of progenitor cells have been identified. The first is the early c‐Myb‐independent primitive wave, which differentiates into macrophages in situ and migrates to the brain as the major source of microglia [8, 11]. The second is the c‐Myb‐dependent erythromyeloid progenitor pathway, in which progenitors differentiate into macrophages in situ; they also migrate into the fetal liver. These progenitors then migrate to developing tissues such as the brain, liver, epidermis, and lungs, where they differentiate into TRMs before birth [11, 12, 13, 14]. In contrast, TRM subsets in the intestine, skin, heart, and pancreas are predominantly maintained postnatally via differentiation of monocyte precursors derived from hematopoietic stem cells [15]. After birth, embryonic macrophages are partially replaced by monocyte‐derived macrophages, depending on the tissue. Under steady‐state conditions, macrophages in the brain, liver, and epidermis are replaced only minimally by hematopoietic stem cell–derived macrophages, preserving yolk sac–derived macrophage populations [11]. The development of early human embryonic macrophages parallels the process observed in mice and zebrafish. A subset of human yolk sac–derived embryonic macrophages appearing at Carnegie stage (CS)11 migrates early to the head and subsequently gives rise to microglia [8]. After CS17, yolk sac–derived myeloid‐biased progenitors produce monocytes capable of migrating to the head and differentiating into macrophages.

There is evidence that TRMs develop during embryogenesis, independent of circulating blood monocytes. Macrophages are first detected in the yolk sac between embryonic days 6.5 and 8.5 (E6.5–E8.5). By E8.5–E10.5, hematopoietic stem cells emerge in the aorta–gonad–mesonephros region, establishing the immune cell lineage. At E10.5, these stem cells migrate to the fetal liver, which becomes the primary hematopoietic organ throughout subsequent embryonic development [16].

### 2.2 Macrophage Diversity

Beyond the well‐known M1 and M2 phenotypes, subsets such as CD169+ and TCR+ macrophages have been identified [17]. These represent specific cell populations induced by particular stimulatory factors within defined disease microenvironments. Alveolar macrophages arise from embryonic progenitors and are gradually replaced by fetal monocytes [18]. Other lung macrophage subsets, including interstitial CD169+ lung‐resident macrophages, transcriptionally and developmentally differ from alveolar macrophages. These tissue‐resident populations originate from the yolk sac, self‐renew, and do not rely on C–C motif chemokine receptor 2 (CCR2)–dependent monocytes for their development or maintenance. They possess anti‐inflammatory properties and help maintain immune homeostasis within lung tissue [19].

#### 2.2.1 M1 and M2 Macrophages

Macrophage polarization encompasses distinct M1 or M2 phenotypes shaped by the surrounding microenvironment. Lymphocyte‐secreted interferon‐γ (IFN‐γ) promotes the differentiation of resting macrophages into “classically activated” or M1 macrophages, which exhibit enhanced antimicrobial activity, regulated phagocytosis, and secretion of proinflammatory cytokines [20]. T helper 1 (Th1) and Th2 classification–related research showed that interleukin (IL)‐4, secreted by Th2 cells, polarizes macrophages into a phenotype distinct from M1 [21]. IL‐4–induced macrophages were defined as an alternative activation state characterized by strong endocytosis of mannosylated ligands, limited expression of MHC‐II antigens, and reduced secretion of proinflammatory cytokines [22]. Further studies identified additional M2 subtypes, including M2b (triggered by immune complexes) and M2c, induced by signals such as IL‐10, transforming growth factor‐β (TGF‐β), and glucocorticoids. These environmental signals drive macrophages to dynamically shift between M1 and M2 states, enabling flexible responses to diverse stimuli.

Regulatory macrophages (Mregs) primarily differentiate from bone marrow precursor cells or peripheral blood monocytes in response to in vivo microenvironmental cues or in vitro stimulatory conditions. These cells represent a macrophage population with immunosuppressive regulatory functions and display surface markers similar to those of M2‐like macrophages. Reported Mreg markers include CD68, CD80, CD150, CD163, CD206, and the human‐specific dehydrogenase/reductase 9 (DHRS9); these markers continue to be refined and updated [23]. Additionally, Mregs express IL‐10 and suppress effector T cells through a contact‐dependent mechanism while promoting the polarization of T cells into regulatory T cells (Tregs) [24]. Mregs also express MHC‐II and CD80, which confer strong antigen‐presenting capabilities [25].

The immunosuppressive capacity of Mregs varies according to the stimuli that induce them. Macrophage colony‐stimulating factor (M‐CSF) and granulocyte–macrophage colony‐stimulating factor (GM‐CSF) can promote the generation of Mregs from bone marrow precursor cells or peripheral blood monocytes. M‐CSF combined with lipopolysaccharide (LPS) and IFN‐γ can also facilitate Mreg induction [26]. Human Mregs generated via M‐CSF and IFN‐γ have been examined in preliminary clinical trials for renal transplantation [27]. Other proinflammatory factors, including IL‐1β, IL‐12, IL‐23, and tumor necrosis factor (TNF)‐α, may contribute to Mreg induction and differentiation under specific conditions. Certain microbial products, such as *Brugia malayi* microfilariae lysate, can induce human monocytes to polarize into Mregs and express PD‐L1 and IL‐10 in vitro [25]. Macrophages that undergo antibody‐dependent phagocytosis of tumor cells can also convert into Mregs, exerting immunosuppressive effects through overexpression of PD‐L1 and indoleamine 2,3‐dioxygenase (IDO) [28]. Furthermore, anti‐TNF agents such as etanercept and adalimumab promote the production of IL‐10–secreting Mregs by activating the signal transducer and activator of transcription 3 (STAT3) pathway [29, 30].

As key components of the immunosuppressive network, Mregs have potential applications in organ transplantation, prevention of rejection, and treatment of autoimmune diseases. Notably, Mregs possess the ability to promote immune tolerance after organ transplantation. In vitro, human Mregs can be generated from CD14+ peripheral blood monocytes stimulated with colony‐stimulating factor 1 (CSF1). These Mregs also inhibit the proliferation of mitogen‐stimulated allogeneic T cells through IFN‐γ–induced IDO and directly interact with T lymphocytes to phagocytose and eliminate activated T cells in an antigen‐specific manner, reducing their numbers [31]. Additionally, Mregs facilitate the development of TIGIT + Foxp3+ Tregs and inhibit dendritic cell maturation, contributing to improved allograft acceptance [24].

In organ transplantation, Mregs can suppress transplant rejection and prolong graft survival. One approach involves directing macrophage polarization toward Mregs in vivo; another strategy relies on the adoptive transfer of in vitro–generated Mregs to establish immune tolerance and suppress rejection in solid organ transplantation. Similar to most cellular therapies, Mregs are induced in vivo or prepared in vitro and subsequently infused into the patient. The infused Mregs inhibit rejection of solid organ grafts, thereby extending graft survival. The ONEmreg12 trial represents an application of Mregs in clinical kidney transplantation; however, Mreg quality and dosing optimization is needed to achieve optimal therapeutic benefit [31]. Importantly, Mreg preparations can be strictly controlled with respect to cellular composition, quality, and dose. Mregs may also be used in combination with conventional immunosuppressive therapies [32, 33]. Overall, clinical applications of Mregs in organ transplantation remain limited; further studies are required to confirm their reliability and therapeutic value.

#### 2.2.2 CD169+ Macrophages

CD169+ macrophages constitute a specialized subset of macrophages [34], primarily distributed in the spleen, lymph nodes, lungs, and bone marrow [35]. These macrophages participate in antigen presentation, regulation of lymphocyte proliferation, induction of inflammatory responses, and defense against viral infections. CD169+ macrophages also appear in the TME, where IFN‐γ can induce their formation. The proinflammatory chemokines they produce can shift the TME from an immunosuppressive state toward an antitumor state [36].

In the lungs, CD169+ macrophages represent a developmentally distinct subset compared with conventional alveolar macrophages. CSF1 induces macrophages to differentiate into lung CD169+ macrophages, which express immunoregulatory genes, suppress excessive inflammatory responses, and maintain lung tissue homeostasis [19]. In the spleen, CD169+ macrophages undergo polarization to a proinflammatory state after sensing *Listeria*; they secrete IL‐10 to mediate immune responses [37]. In bone, CD169+ macrophages—functionally distinct from osteoclasts—provide essential anabolic support to osteoblasts during bone homeostasis and repair [38]. Furthermore, CD169+ macrophages exert anticancer effects within the TME. Studies have shown that low‐dose IFN‐α causes macrophage polarization into the CD169+ phenotype, enhancing their phagocytic capacity and increasing CD8+ T cell activation, thereby inhibiting human hepatocellular carcinoma (HCC) [39].

#### 2.2.3 TCR+ Macrophages

In addition to the macrophage types described above, a subset of macrophages expresses the TCR. These cells—mainly present at inflammatory sites in healthy individuals—are associated with the development of inflammatory diseases. Early studies demonstrated that recombinant immune receptors for TCRαβ are detectable in peripheral blood monocytes, TRMs, and macrophages located at inflammatory lesions. Macrophages from healthy individuals can be activated by mycobacteria and subsequently express clusters of restricted TCR Vβ repertoires. TCRαβ+ macrophages are detectable in tuberculous granulomas, where they stimulate the expression and secretion of C–C motif chemokine ligand 2 (CCL2) and enhance related phagocytic functions [40]. In addition to TCRαβ+ macrophages found in tuberculosis lesions, TCRγδ+ macrophages can be detected in atherosclerosis and bacterial meningitis lesions, representing an immune response elicited after bacterial infection [41]. Researchers have also identified individual‐specific TCRαβ+ macrophages within the TME of breast cancer [42]. Furthermore, scRNA‐seq has revealed a TCR+ macrophage subset with enhanced phagocytic capacity in the TME of head and neck squamous cell carcinoma, which may participate in the TCR signaling pathway and exhibit functional associations with T cells [43].

#### 2.2.4 TAMs

TAMs of various developmental origins exhibit distinct functions and exert diverse effects on tumor initiation, proliferation, and migration. Studies in multiple human and mouse tumor models have shown that TAMs arise not only from the local proliferation of TRMs of embryonic origin but also from monocytes and monocyte‐related myeloid‐derived suppressor cells. The TME of solid tumors contains macrophages from various sources; the majority originate from recruited peripheral blood monocytes, and a minority are derived from TRMs [33, 34]. In addition to these two macrophage lineages, TAMs have been identified across multiple tumor types [44]. TAMs represent the most abundant innate immune cell population in the TME and typically exhibit extensive infiltration, influencing tumor development through distinct immune states [45]. In most solid malignancies, tumor cells actively recruit myeloid monocytes via cytokines, chemokines, and exosomes, leading to substantial infiltration of immunosuppressive macrophages [46, 47]. Environmental factors within the TME may induce TAMs to acquire antitumor M1‐like properties and secrete proinflammatory cytokines. However, the predominant M2‐like macrophages facilitate metastasis, enhance immunosuppression, and promote tumor cell proliferation and immune escape [48]. The TME recruits myeloid monocytes through factors including CSF1, IL‐1β, and chemokines such as CCL2, CCL5 [49], and C–X–C motif chemokine ligand (CXCL)12. Thus, regardless of whether TAMs originate from monocytes or TRMs, recruitment into the TME occurs through diverse cytokines, chemokines, and tumor‐derived exosomes; it is influenced by tumor type. TAMs can be induced to polarize into various immune phenotypes in response to specific stimuli, thereby shaping tumor progression [50].

## 3 Regulatory Mechanisms of TAMs: Polarization and Functional Plasticity

TAMs in the TME are regulated by a complex signaling network within the TME, enabling macrophages to switch between different functional states. This section first outlines the key signaling pathways by which TAMs switch between proinflammatory M1‐like and immunosuppressive M2‐like phenotypes. We then explore how molecular, metabolic, and epigenetic mechanisms help stabilize these different states. Finally, we link these regulatory networks to the roles of TAMs in angiogenesis, immunosuppression, and tumor progression, highlighting the central importance of macrophage plasticity in cancer biology.

### 3.1 Macrophage Polarization in the TME

Macrophage polarization is primarily influenced by three major regulatory factors: extrinsic stimuli, intrinsic developmental pathways, and the local tissue environment. It depends on the specific spatial context of the cell and involves multiple regulatory mechanisms [51, 52]. Distinct surface marker profiles emerge after monocytes differentiate into macrophages and undergo polarization, resulting in increased responsiveness to diverse secreted factors. Secreted factor–mediated macrophage polarization is also shaped by mechanisms such as hypoxia and intratumoral lactate production [53, 54].

M1 macrophages are proinflammatory cells that undergo polarization via IFN‐γ [55], LPS [56], TNF‐α, and GM‐CSF secreted by Th1 cells, cytotoxic T lymphocytes, and NK cells [57]. During classical activation of M1 macrophages, multiple signaling pathways coordinate the polarization state and resulting immune functions. First, the Toll‐like receptor (TLR)4–MyD88–nuclear factor (NF)‐κB signaling axis, a central mechanism in response to LPS stimulation, is initiated when TLR4 recognizes pathogen‐associated molecular patterns. MyD88 then mediates activation of IKK through the IL‐1 receptor–associated kinase (IRAK)–TNF receptor–associated factor 6 (TRAF6) complex, resulting in IκB degradation and NF‐κB nuclear translocation, which drives expression of proinflammatory cytokines such as TNF‐α, IL‐6, IL‐1β, and inducible nitric oxide synthase (iNOS) [58, 59]. Second, the IFN‐γ–Janus kinase (JAK)–STAT1 pathway plays a critical role in Th1 immunity. IFN‐γ binding to its receptor activates JAK1/2, inducing STAT1 phosphorylation and nuclear translocation, which promotes the expression of inflammatory mediators such as IL‐12 and CXCL10 and enhances CD4+ T‐cell responses. GM‐CSF also amplifies M1 polarization by activating STAT5 [60, 61]. Additionally, under TLR7/9 stimulation, the transcription factor interferon regulatory factor 5 (IRF5) directly targets promoters of inflammatory genes by interacting with RelA (p65), substantially upregulating IL‐12p40 and TNF‐α while suppressing M2‐related genes such as arginase 1 (Arg1), thus maintaining M1 lineage stability [62]. Nuclear factor of activated T cells 5 (NFAT5), a core transcriptional regulator under cellular stress, enhances expression of iNOS and suppressor of cytokine signaling 3 (SOCS3) in hypertonic environments; it also supports macrophage and T‐cell proinflammatory functions through synergy with STAT1 and NF‐κB [63, 64]. Polarized M1 macrophages induce the expression and secretion of TNF‐α, IFN‐γ, IL‐1β, IL‐12, IL‐18, IL‐6, nitric oxide, CCR7, iNOS, and SOCS3, all of which contribute to type I immune responses. They also express high levels of MHC‐II molecules, costimulatory molecules (CD80 and CD86), and various TLRs. These features confer strong antigen‐presenting capacity, enable activation of Th1 responses, and inhibit tumor growth [15, 52].

At the metabolic level, M1 TAMs primarily rely on aerobic glycolysis and interrupted tricarboxylic acid cycles to generate adenosine triphosphate and metabolic intermediates that support their proinflammatory functions [65]. The glycolytic intermediate succinate stabilizes hypoxia inducible factor‐1α (HIF‐1α) expression and promotes the synthesis of proinflammatory factors such as IL‐1β and TNF‐α [66]. In parallel, iNOS utilizes arginine to produce nitric oxide, which exerts cytotoxic effects and competes with Arg1 for substrate availability, thus inhibiting the M2 program [67]. In lipid metabolism, cholesterol metabolism in M1 macrophages—such as through acyl‐CoA:cholesterol acyltransferase 1 (ACAT1) inhibition—has shown close associations with antitumor activity [68]. ACAT1 inhibition blocks cholesterol esterification, resulting in the accumulation of free cholesterol, which enhances TLR4–NF‐κB signaling and suppresses peroxisome proliferator–activated receptor (PPAR)γ activity. This shift promotes M1 polarization and proinflammatory cytokine production, considerably strengthening antitumor immune responses [69]. M1 polarization is also regulated by epigenetic factors. For example, miR‐155 enhances STAT1 and NF‐κB activity while suppressing SOCS1 expression, thus amplifying inflammatory responses [70]. H3K4me3 (trimethylation of lysine 4 on histone H3) enrichment at the TNF‐α and IL‐6 promoters increases their transcription [71]. Finally, NFAT5 synergizes with STAT1 to activate iNOS and SOCS3 transcription, further enhancing antigen‐presenting capacity [63].

In contrast to the proinflammatory responses dominated by M1 macrophages, M2 macrophages primarily exert immunosuppressive and tissue‐repairing functions. Their polarization is typically driven by cytokines such as IL‐4, IL‐13, IL‐10, CSF1, and TGF‐β. These signals remain continuously active in chronic inflammation, parasitic infection, and the TME, directing macrophage differentiation toward the M2 lineage. Activation of the JAK–STAT pathway, particularly STAT6, plays a central role in this process. IL‐4 and IL‐13 binding to their coreceptors—IL‐4Rα with the γ‐chain or IL‐13Rα1—activates JAK1/3, which phosphorylates STAT6 and enables its nuclear translocation to induce transcription of classic M2 markers such as Arg1, found in inflammatory zone 1 (Fizz1), chitinase‐like protein 3 (Ym1), and mannose receptor C‐type 1 (MRC1). TGF‐β also promotes the expression of regulatory mediators such as IL‐10 and tissue inhibitor of metalloproteinases (TIMP)‐3 through the Smad2/3 signaling axis, whereas IL‐10 establishes a positive feedback loop via STAT3 to further amplify M2 signaling. At a broader transcriptional level, PPARγ and Krüppel‐like factor 4 (KLF4) stabilize the M2 program by maintaining the anti‐inflammatory phenotype and suppressing M1‐associated pathways such as NF‐κB and IRF5. Moreover, mechanistic target of rapamycin complex 2 (mTORC2)–mediated metabolic reprogramming promotes fatty acid oxidation and mitochondrial respiration, providing metabolic support for M2 macrophage homeostasis and function [69, 72, 73, 74]. Functionally, M2 macrophages regulate immune response intensity and limit tissue damage via secretion of IL‐10, TGF‐β, and Arg1. They also support tumor progression across cancer types by promoting angiogenesis, immune escape, and extracellular matrix remodeling [75]. Surface molecules such as CD206 (MRC1), CD163, PD‐L1, and macrophage scavenger receptor 1 are strongly expressed and serve as key markers of immunosuppressive TAMs. CD206 mediates transventricular macrophage migration and microglial differentiation during embryonic development; it also regulates cooperative interactions among immune cells in adult tumor models. The activation or inhibition of CD206 may therefore have substantial effects on the TME [76, 77].

M2 TAMs display a clear contrast to M1 cells in terms of metabolic and epigenetic regulation. Their function depends on mitochondria‐driven metabolic programs such as fatty acid oxidation and oxidative phosphorylation (OXPHOS), rather than glycolysis [78]. Under IL‐4/IL‐13 stimulation, the STAT6 signaling pathway is activated, leading to the upregulation of mitochondrial function and fatty acid oxidation–related genes. This upregulation enables fatty acids to enter mitochondria for β‐oxidation, producing NADH and FADH_2_ to fuel the electron transport chain and continuously generate adenosine triphosphate, thus sustaining long‐term immunosuppressive activity [79]. Concerning amino acid metabolism, M2 TAMs characteristically overexpress Arg1, which converts arginine into ornithine. Ornithine subsequently generates proline and polyamines, contributes to collagen synthesis and extracellular matrix remodeling, and promotes angiogenesis, supporting tissue repair and tumor growth [78]. M2 TAMs also exhibit active cholesterol metabolism; enhanced ACAT1 activity drives cholesterol esterification and storage in lipid droplets, maintaining membrane homeostasis and reinforcing the immunosuppressive phenotype by supplying substrates for signaling molecules [80]. At the epigenetic level, M2 polarization depends on STAT6‐mediated chromatin remodeling, characterized by H3K4me3 enrichment and H3K27me3 (trimethylation of lysine 27 on histone H3) reduction in the promoter regions of M2 marker genes such as Arg1 and MRC1, enhancing their transcription [79, 81]. Furthermore, noncoding RNAs contribute to maintenance of the M2 phenotype. For example, miR‐146a suppresses inflammatory gene expression by inhibiting NF‐κB signaling, stabilizing the immunosuppressive state [82]. Epigenetic modulation holds therapeutic potential; upregulation of miR‐7083‐5p can induce repolarization of M2 TAMs toward an M1 phenotype, restoring their proinflammatory and antitumor functions [83].

**FIGURE 1:**
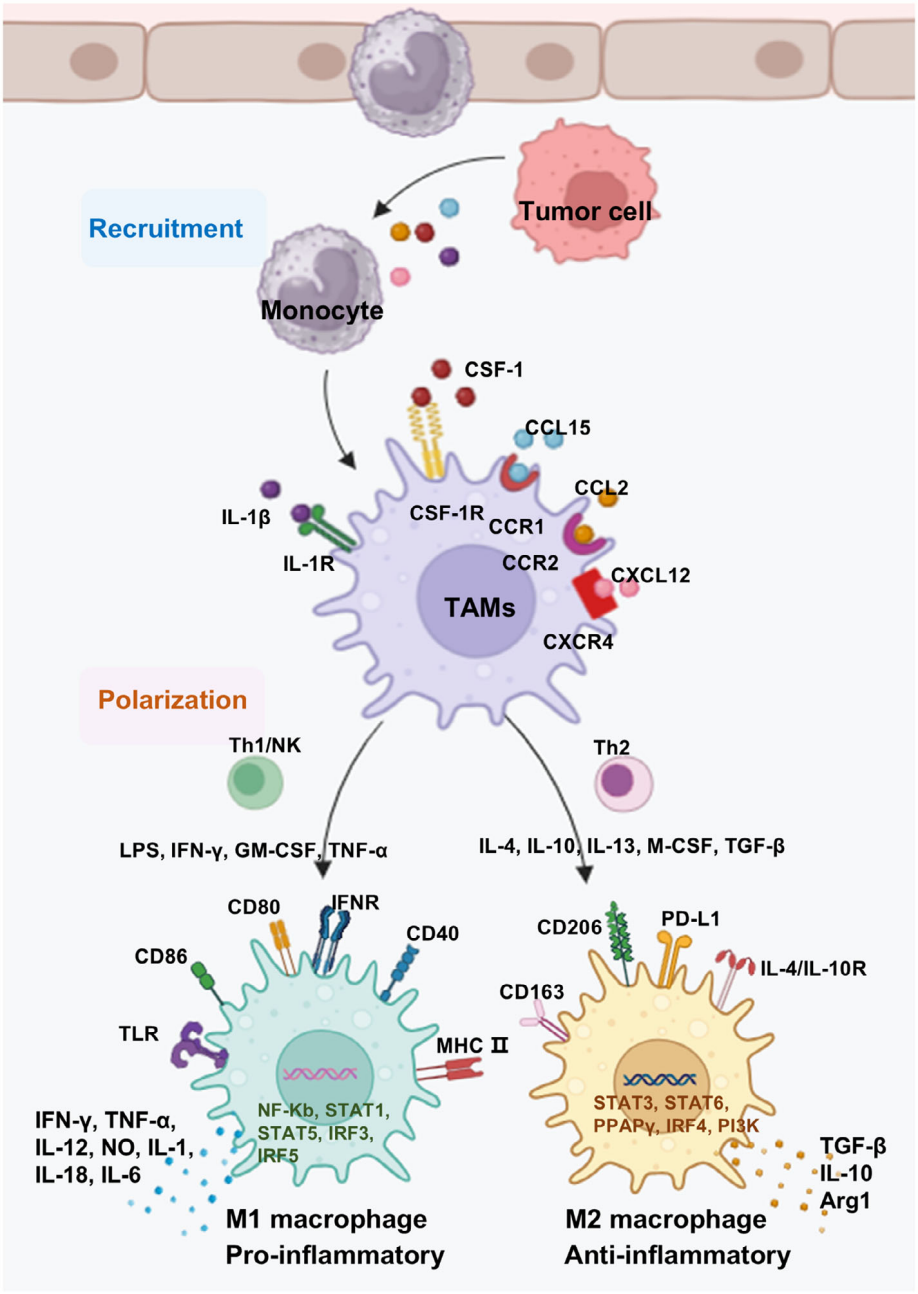
Recruitment and polarization of TAMs. Monocytes are recruited to the TME and differentiate into TAMs in response to CSF1, CCL15, CXCL12, IL‐1β, and other factors secreted by tumor cells. TAMs polarize toward M1 via stimulation by LPS, IFN‐γ, GM‐CSF, and TNF‐α, leading to the secretion of proinflammatory mediators. TAMs polarize toward M2 under the influence of IL‐4, IL‐10, IL‐13, M‐CSF, and TGF‐β, producing anti‐inflammatory factors such as TGF‐β, Arg1, and IL‐10. Arg1, arginase 1; CSF1, colony‐stimulating factor 1; GM‐CSF, granulocyte–macrophage colony‐stimulating factor; IFN‐γ, interferon‐gamma; IL, interleukin; LPS, lipopolysaccharide; TAMs, tumor‐associated macrophages; TGF‐β, transforming growth factor‐beta; TME, tumor microenvironment; TNF‐α, tumor necrosis factor‐alpha.

Peripheral blood monocytes can be recruited into the TME and differentiate into TAMs in response to tumor cell–secreted recruitment factors such as CSF1, CCL2, and CXCL12. TAMs may subsequently polarize into M2‐like TAMs under the influence of additional regulatory factors, promoting tumor cell proliferation, migration, and angiogenesis [84]. TAMs share substantial similarity with M2 macrophages and express classical M2 biomarkers such as CD163, macrophage scavenger receptor 1, and CD206. Fibroblast growth factor 2 (FGF2)—secreted by cancer‐associated fibroblasts (CAFs)—exerts protumor effects, and serine protease 23 enhances TAM infiltration via FGF2 [85]. Additional regulators of macrophage polarization include microRNAs, phosphatases, and metabolic enzymes (Figure 1).

### 3.2 Functions of TAMs in the TME

**FIGURE 2:**
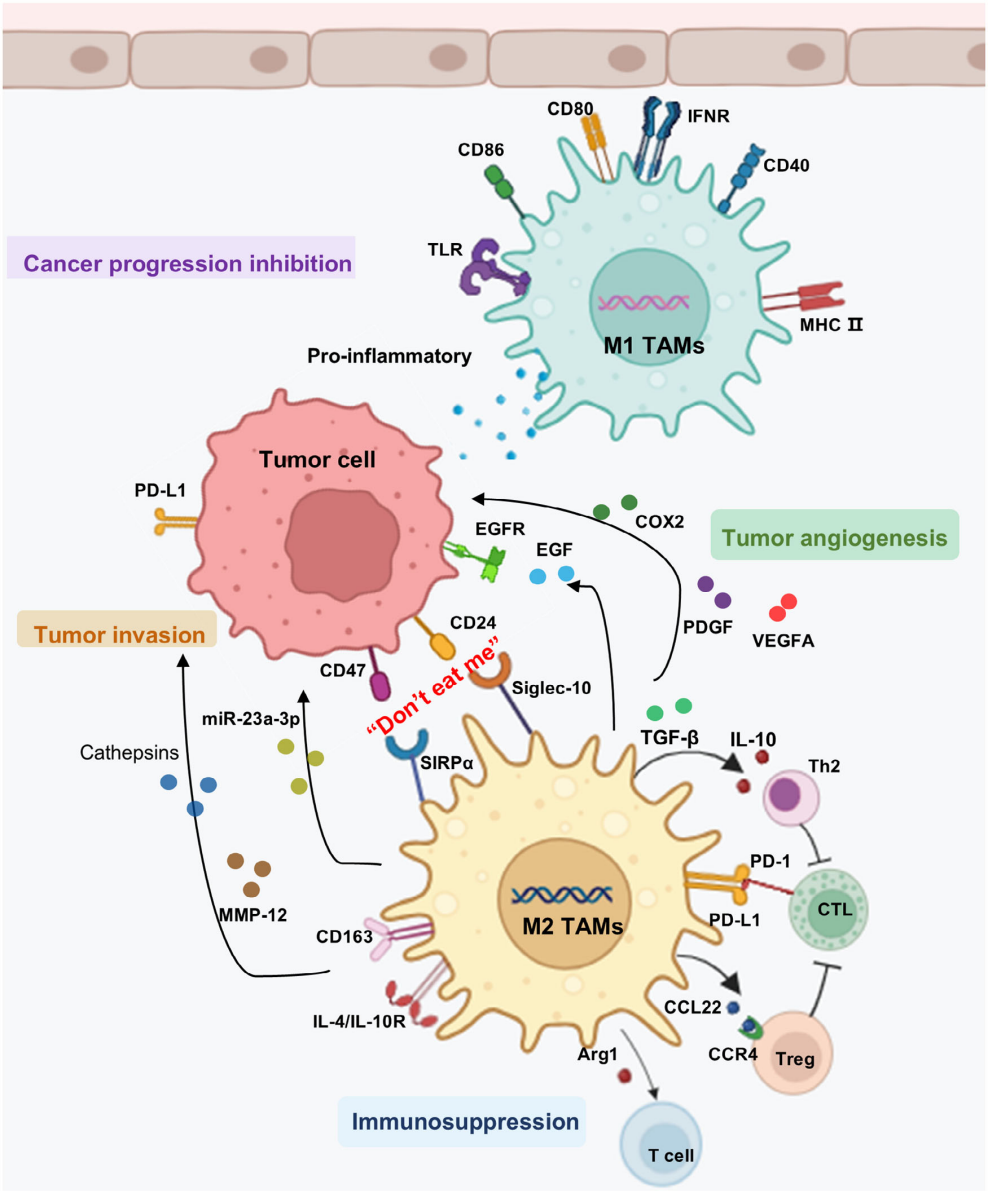
Effects of TAMs on tumor progression within the TME. CD24 and CD47 on tumor cells bind to Siglec‐10 and SIRPα on M2 TAMs, enabling tumor cells to evade macrophage phagocytosis through the “don't eat me” pathway. M2 TAMs promote tumor migration and invasion by secreting MMP‐12 and cathepsins, along with reduction of EMT. They also inhibit inflammatory cell function through anti‐inflammatory mediators, creating an immunosuppressive TME. Additionally, M2 TAMs support tumor angiogenesis by secreting PDGF, VEGFA, and EGF. In contrast, M1 TAMs release proinflammatory factors that counteract tumor progression. EGF, epidermal growth factor; EMT, epithelial–mesenchymal transition; MMP, matrix metalloproteinase; PDGF, platelet‐derived growth factor; TAMs, tumor‐associated macrophages; TME, tumor microenvironment; VEGFA, vascular endothelial growth factor A.

In most TMEs, TAMs predominantly exist as M2‐like cells and promote tumor cell proliferation, angiogenesis, and the formation of an immunosuppressive microenvironment that facilitates tumor metastasis. TAMs interact with tumor cells, as well as the broader TME. In addition to undergoing polarization and repolarization in response to microenvironmental signals, TAMs influence the TME through the expression of specific surface markers and the secretion of growth factors and other bioactive molecules (Figure 2).

TAMs secrete vascular endothelial growth factor (VEGF), cyclooxygenase‐2, platelet‐derived growth factor (PDGF), and exosomes to promote tumor angiogenesis. One study [86] revealed that in the HCC microenvironment, M2 TAMs release exosomes containing miR‐23a‐3p, which target HCC cells and cause them to secrete additional recruitment factors, further increasing M2 TAM infiltration. miR‐23a‐3p also targets phosphatase and tensin homolog (PTEN) and tight junction protein 1, promoting epithelial–mesenchymal transition and enhancing tumor migration. In ovarian cancer, embryonic TAMs expressing CD163 and T‐cell immunoglobulin and mucin‐domain containing molecule 4 (Tim4) produce high levels of IL‐6 and erythropoietin, activating STAT3 to promote tumor progression and invasion [87]. In lung cancer, TAMs secrete IL‐10, which supports tumor cell proliferation through the JAK1/STAT1/NF‐κB/Notch1 pathway [88]. In the prostate cancer TME, tumor‐derived PDGF acts on TAMs via PDGFR, inducing their polarization; these polarized TAMs then secrete TGF‐β1, further promoting prostate cancer cell growth [89]. Under the hypoxic conditions generated by rapid tumor cell proliferation, TAMs predominantly shift toward the M2 phenotype and upregulate IL‐10, VEGF, and HIF‐1α, contributing to cancer metastasis and an increasingly immunosuppressive TME. Additionally, three‐dimensional coculture models that simulate immunosuppressive conditions containing IL‐10 and CXCL1 have demonstrated enhanced cancer cell migration and progression [84].

TAMs also promote tumor invasion and metastasis by secreting matrix metalloproteinases (MMP‐2, MMP‐7, MMP‐9, and MMP‐12) [90], cathepsins [91], and urokinase‐type plasminogen activator, which degrade the extracellular matrix. In ovarian cancer, TAM‐derived extracellular vesicles upregulate GATA‐binding protein 3 (GATA3) by activating the CD24/sialic acid‐binding IgG‐like lectin 10 (Siglec‐10) axis, promoting the immune escape of tumor cells [92].

Within the TME, TAMs can reduce T‐cell proliferation and enhance Treg induction by secreting various factors and engaging multiple surface molecules, including high levels of IDO and CD40/CD40L [93, 94, 95]. TAM‐derived TGF‐β promotes Treg recruitment by inducing CCL22 expression in TAMs via c‐Fos activation [96]. Conversely, Tregs regulate TAM expansion and function. Tregs enhance TAM proliferation and immunosuppression through TGF‐β–dependent mechanisms and the PD‐L1 pathway [97]. Furthermore, Tregs secrete IL‐8, which induces TAMs to produce TGF‐β, further reinforcing the immunosuppressive TME [96]. TAMs also participate in antigen presentation. CD206+ TAMs function as cross‐presenting cells and strongly express CD86, inducible T‐cell costimulator ligand (ICOSLG), and C‐type lectin receptor 4A (CLEC4A), promoting antigen cross‐presentation and antigen‐specific CD8+ T‐cell activation, thereby reducing tumor burden [98].

Metabolic and epigenetic regulation plays a central role in the immunosuppressive and tumor‐promoting functions of TAMs. High expression of 3‐oxoacid CoA transferase 1 (OXCT1) drives ketone body metabolism and leads to succinate accumulation, promoting TAM polarization toward an immunosuppressive phenotype. In HCC, OXCT1 inhibition substantially enhances CD8+ T‐cell–mediated antitumor immunity [99]. TAMs also suppress effector T‐cell activation and proliferation by degrading arginine in the TME via high Arg1 expression, weakening TCR–CD3ζ signaling—a major mechanism of immunosuppression confirmed in pancreatic cancer [100]. Additionally, M2‐like TAMs demonstrate the highest single‐cell glucose uptake capacity in the TME [89]. TAMs competitively consume glucose, enabling O‐linked N‐acetylglucosamine transferase to glycosylate Ser210 of the lysosomal protease cathepsin B with O‐GlcNAc, promoting formation of mature cathepsin B in TAMs and the TME, thus driving tumor metastasis and chemotherapy resistance in mice [101]. Lactate accumulation in the TME also plays a critical regulatory role. Beyond its metabolic origin, lactate serves as a substrate for epigenetic modification. Lactate‐derived lactyl‐CoA mediates histone lysine lactylation at residues such as H3K18 and H4K12, activating transcription of M2‐associated TAM genes (e.g., Arg1 and MRC1) [102, 103]. This modification cooperates with the p300 acetyltransferase complex to regulate histone and nonhistone lactoylation, amplifying immunosuppressive activity [104]. Recent studies have shown that in tumors such as glioblastoma and HCC, lactate‐induced histone lactylation greatly enhances the immunosuppressive function of TAMs, driving continuous IL‐10 and TGF‐β secretion, suppressing effector T‐cell responses, and contributing to immunotherapy resistance [105, 106]. The lactate–lactylation axis intersects with other metabolic–epigenetic networks, including fatty acid synthesis and DNA methylation, to reinforce TAM‐mediated immunosuppression in the TME [107, 108].

## 4 TAM‐Based Therapeutic Strategies

**FIGURE 3:**
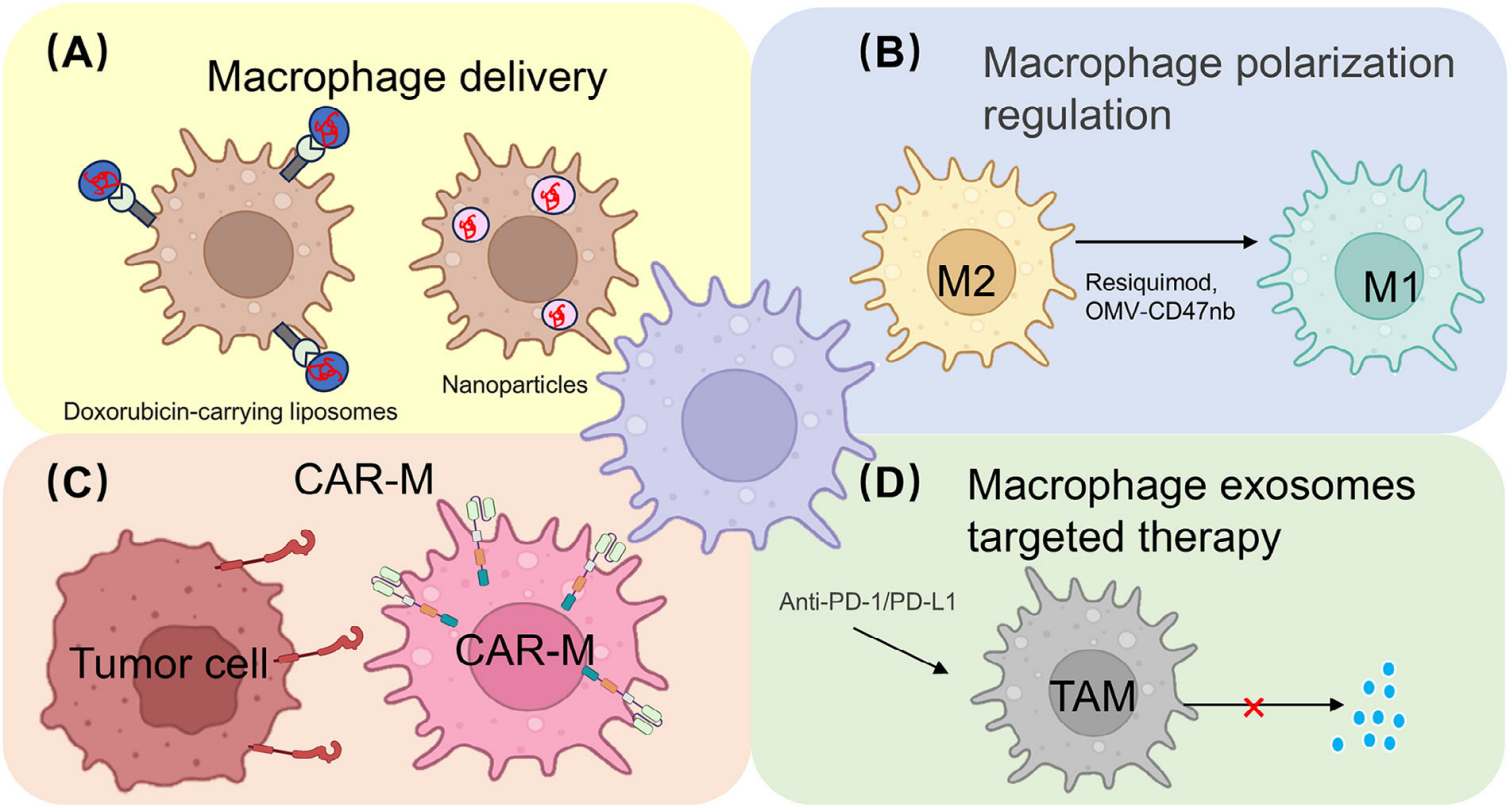
Macrophage‐based strategies in cancer therapy. (A) Macrophages as delivery vehicles: Macrophages can serve as carriers for liposomes or nanoparticles loaded with chemotherapeutic agents such as Dox. (B) Regulation of macrophage polarization: Exogenous factors can reprogram TAMs from an M2 phenotype to a proinflammatory M1 phenotype. (C) CAR‐Ms: Through genetic engineering, macrophages are modified to recognize and target tumor cells. CAR‐Ms have been developed in three generations: (1) First‐generation CAR‐Ms primarily recognize tumor‐specific antigens and mediate phagocytosis, with additional molecules such as CD147 enabling penetration of the tumor extracellular matrix. (2) Second‐generation CAR‐Ms are optimized to improve antigen presentation and promote T‐cell activation, strengthening the connection between innate and adaptive immunity. (3) Third‐generation CAR‐Ms incorporate immune‐modulatory genes such as IFN‐γ and TLR4 to reprogram M2 TAMs toward the M1 phenotype and enhance antitumor efficacy. (D) Macrophage‐derived exosomes: TAMs secrete PD‐L1–containing exosomes. Methods that target TAMs or their exosomes can inhibit tumor progression. CAR‐Ms, chimeric antigen receptor macrophages; Dox, doxorubicin; IFN‐γ, interferon‐gamma; PD‐L1, programmed death ligand 1; TAMs, tumor‐associated macrophages; TLR4, Toll‐like receptor 4.

As the crucial regulatory role of TAMs in tumor progression becomes increasingly evident, use of their plasticity for therapeutic benefit has emerged as a major research focus. This section outlines macrophage‐centered therapeutic strategies. We first examine how TAM plasticity can be used to repolarize immunosuppressive M2‐like macrophages into proinflammatory M1‐like cells, restoring antitumor function. Next, we highlight CAR‐Ms, a next‐generation cell therapy designed to enhance phagocytosis, antigen presentation, and immune activation within the TME. Finally, we summarize macrophage‐mediated drug delivery and exosome‐based strategies that exploit the innate tumor‐homing capacity of macrophages to achieve targeted therapy and modulation of the immune microenvironment (Figure 3). Collectively, these approaches represent macrophage‐centered cancer immunotherapies with robust clinical potential.

### 4.1 TAM Plasticity and Tumor Therapy

Macrophages exhibit functional plasticity in response to microenvironmental signals. Clinical studies have shown that M1 macrophages can be repolarized into M2 macrophages, or M2 macrophages can transition to M1 macrophages, depending on disease context. For instance, to inhibit chronic inflammatory diseases such as atherosclerosis and rheumatoid arthritis, M1 macrophage repolarization into M2 cells can effectively suppress inflammation [109]. Conversely, in oncology, a key therapeutic strategy comprises repolarizing M2‐like macrophages into M1‐like macrophages to restore their proinflammatory and antitumor activities [110, 111].

Therapeutic approaches targeting TAMs in cancer include inhibition of TAM recruitment, depletion of TAMs, and reprogramming of M2‐like TAMs into M1‐like TAMs. TAMs are highly plastic and shift between phenotypes in response to endogenous or exogenous cues. Signals produced by tumor cells and immune cells within the TME polarize recruited macrophages toward M1‐like or M2‐like states. In addition to the inhibition of TAM recruitment, strategies that leverage macrophage plasticity aim to reprogram tumor‐promoting M2‐like TAMs into M1‐like TAMs by introducing exogenous modulators or enhancing endogenous TME‐derived signals. The resulting M1‐like macrophages secrete proinflammatory mediators that inhibit tumor progression. Therapeutic modulation from multiple angles can introduce exogenous signals that repolarize M2‐like TAMs into M1‐like cells, thus suppressing tumor cell proliferation, migration, and angiogenesis.

Cell morphology studies have shown that M1 macrophages are typically round, whereas M2 macrophages exhibit an elongated shape. Under in vitro culture conditions, when macrophages adopt an elongated morphology, inflammatory cytokine secretion decreases, and the cells become more responsive to IL‐4 and IL‐13, promoting polarization toward the M2 phenotype. Inhibiting the tendency of macrophages to elongate slows or prevents their polarization into M2 macrophages [112]. Based on this observation, cytoskeletal microtubule targeting with the microtubule inhibitor cabazitaxel activates NF‐κB signaling and directly promotes polarization toward the M1 state, thus enhancing macrophage‐mediated programmed cell removal and improving clearance of triple‐negative breast cancer cells [113]. Similarly, vinblastine destabilizes microtubules and induces TAM polarization toward the M1 phenotype, further activating the NF‐κB pathway and Cyba‐dependent reactive oxygen species production, ultimately enhancing phagocytic function [114].

Studies have also shown that M2 macrophages exhibit greater plasticity and can be readily reprogrammed into inflammatory M1 macrophages. Immunometabolic research has demonstrated that inhibition of mitochondrial OXPHOS in M1 macrophages restricts repolarization toward the M2 phenotype. Nitric oxide production helps sustain M1‐like behavior; thus, modulation of mitochondrial OXPHOS can maintain either proinflammatory or anti‐inflammatory activity in TAMs [115]. Furthermore, the glycopeptide dCP1, isolated from *Codonopsis pilosula*, substantially upregulates glycolysis‐related genes and genes associated with M1 polarization. It drives the reprogramming of M2‐like macrophages toward an M1 phenotype, suggesting utility as a macrophage polarization modulator and a therapeutic agent for cancer treatment [116]. In contrast, the long noncoding RNA NR_109 is strongly expressed in CD163+ TAMs in gastric and breast cancer tissues, where it activates c‐Myc transcription to promote M2 polarization. Therefore, NR_109 knockdown may serve as an immunotherapeutic strategy to inhibit M2 polarization [117].

In clinical applications, inhibitors of the CSF1/CSF1R pathway are used as immunotherapeutic agents to promote repolarization of M2 TAMs into antitumor M1 TAMs. CSF1R small interfering RNA (siRNA) and other inhibitors have successfully induced M1 repolarization in multiple tumor models, including colorectal cancer [118], high‐grade serous fallopian tube ovarian cancer [119], and follicular lymphoma [120].

TAMs also influence tumor progression by modulating T‐cell activity. Specifically, TAMs inhibit the cytotoxic function of CD8+ T cells through class I phosphoinositide 3‐kinase (PI3K) signaling and undergo polarization to an M2 phenotype that promotes tumor progression. Nanomicelles have been developed to deliver the PI3K‐γ inhibitor NVP‐BEZ235 together with CSF1R siRNA, effectively repolarizing M2 TAMs into M1 TAMs in pancreatic ductal adenocarcinoma models [121]. Combination therapy using CSF1/CSF1R inhibitors and PD‐L1 blockade further enhances repolarization of M2 TAMs into M1 TAMs and increases CD8+ T‐cell infiltration, considerably improving antitumor efficacy [122].

During chemotherapy‐induced endocytosis, TAMs often shift toward a tumor‐promoting M2‐like phenotype. Rather than blocking endocytosis, the induction of M1 polarization during this process is a promising therapeutic approach. In breast cancer, administration of the immunomodulator thymosin α‐1 (Tα‐1) results in its binding to phosphatidylserine on apoptotic tumor cells. After uptake by TAMs, Tα‐1 activates TLR7/MyD88–Src homology 2 domain–containing inositol‐5‐phosphatase 1 (SHIP1) signaling, inhibits dephosphorylation of TANK‐binding kinase 1, and reduces IL‐10 secretion, thereby suppressing M2 polarization [123]. Combined treatment with Tα‐1 and epirubicin further increases CD8+ T‐cell infiltration and suppresses tumor cell proliferation in vivo.

Similarly, the TLR7/8 agonist resiquimod (R848) has been shown to induce repolarization of M2 TAMs [124, 125]. Multiple groups have developed targeted delivery systems combining multiple agents that modulate the suppressive immune microenvironment—such as poly(lactide‐co‐glycolide) (PLGA)–doxorubicin, temozolomide, and R848—to enable synergistic chemoimmunotherapy [126, 127, 128]. CD5 molecule‐like (CD5L), a secreted glycoprotein mainly produced by macrophages and other immune cells, regulates M2 macrophages and inhibits M1 macrophage function. In vivo studies with a novel anti‐CD5L monoclonal antibody demonstrated its ability to modulate TME immunosuppression and induce TAM polarization into the antitumor M1 phenotype, thereby enhancing inflammatory cytokine production and promoting antitumor effects [129].

### 4.2 Engineered Therapy: CAR‐Ms

TAMs infiltrating the TME are mainly monocytes recruited by tumor‐derived signaling molecules [48, 130]. TAMs extensively accumulate within tumors and possess strong phagocytic activity, antigen‐presenting capability, and innate tumor‐infiltrating properties. Therapeutic strategies targeting TAMs include regulation of TAM polarization, inhibition of TAM recruitment, and genetic engineering of TAMs. However, direct therapeutic manipulation of TAMs is limited by the heterogeneous developmental origins and dynamic state transitions of TAMs within and across tumor types [45]. Current macrophage‐targeted cancer therapies primarily focus on engineering macrophages to enhance their phagocytic activity, enable specific tumor targeting, or activate T cells to suppress tumor development [131].

CAR‐T and CAR‐NK cell therapies are already well established in clinical practice. Although CAR‐T therapy has demonstrated efficacy in hematologic malignancies, its infiltration into solid tumors is limited; CAR‐T cells often become inactivated within the TME. Moreover, CAR‐T treatment increases the proportion of M2 TAMs in the TME, leading to enhanced immunosuppression. These observations prompted researchers to explore macrophages—cells with natural tumor infiltration capacity and robust phagocytic function—as alternative engineered immune effectors, leading to the development of CAR‐Ms. In recent years, CAR‐M design has been progressively optimized to enhance safety and therapeutic efficacy [132, 133]. Compared with CAR‐T and CAR‐NK therapies, CAR‐M therapy offers distinct advantages because macrophages can penetrate solid tumors more effectively and sustain infiltration within the TME. Structurally, CAR‐M constructs share similarities with CAR‐T constructs. CAR‐Ms typically include extracellular domains (e.g., CD19 or human epidermal growth factor receptor 2 [HER2] single‐chain variable fragment), hinge regions, transmembrane domains (e.g., CD8 or CD147), and intracellular domains such as CD3ζ or FcγR that mediate activation and phagocytosis. Delivery systems—including adenoviral vectors, plasmid‐based approaches, and nanoparticle‐encapsulated mRNA—continue to be refined. CAR‐M therapies can be generated from multiple cellular sources, including peripheral blood monocytes, induced pluripotent stem cells (iPSCs), and monocytic cell lines such as THP‐1. Preclinical tumor models consistently demonstrate that CAR‐Ms suppress tumor progression [132]. In summary, continuous advancements in CAR‐M structure, manufacturing methods, and engineering strategies have accelerated the development of this approach into a promising next‐generation modality for cancer immunotherapy.

#### 4.2.1 CAR‐M Structure and Mechanism

**FIGURE 4:**
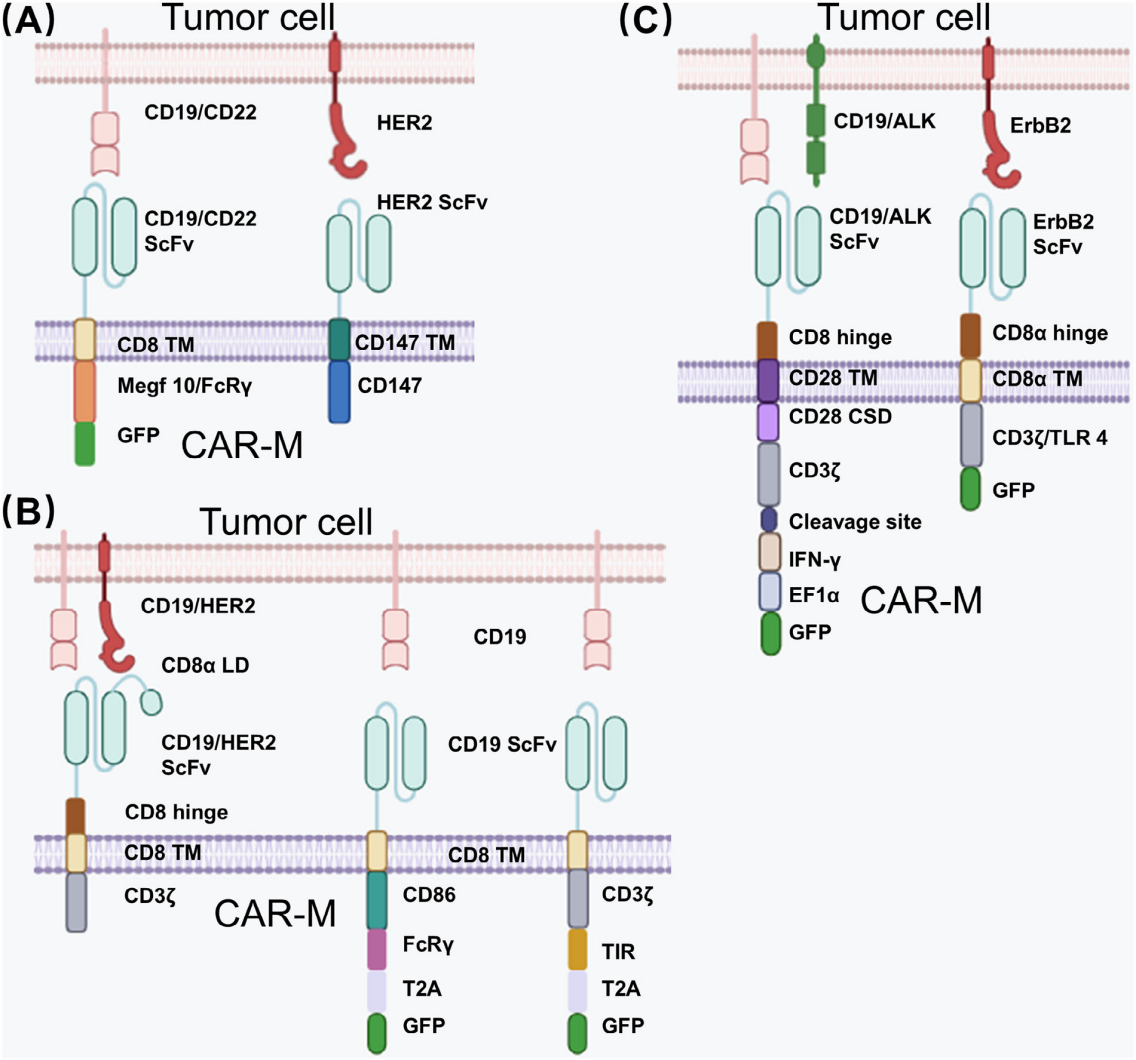
Three generations of CAR‐M structures designed for different tumor targets. CAR‐Ms consist of three major components: an extracellular domain that recognizes tumor cells, a transmembrane domain, and an intracellular domain that initiates downstream signaling pathways. (A) First‐generation CAR‐Ms are designed to recognize tumor‐specific antigens and mediate phagocytosis. CD147 facilitates penetration of the tumor ECM. (B) Second‐generation CAR‐Ms are engineered to enhance tumor‐associated antigen presentation and promote T‐cell activation. (C) Third‐generation CAR‐Ms use in vivo nonviral vector–based reprogramming and incorporate IFN‐γ and TLR4 genes to repolarize M2 macrophages toward the proinflammatory M1 phenotype, improving antitumor efficacy. CAR‐Ms, chimeric antigen receptor macrophages; ECM, extracellular matrix; IFN‐γ, interferon‐gamma; TLR4, Toll‐like receptor 4.

CAR‐M therapy is mainly designed to reprogram M2 macrophages into proinflammatory M1 macrophages and to promote the phagocytosis of targeted tumor cells. Additional mechanisms include antigen presentation and enhanced T‐cell infiltration. The design and structural evolution of CAR‐M constructs can be broadly categorized into three generations, each optimized for distinct functional and therapeutic objectives (Figure 4). These developments reflect the rationale for selecting macrophages as therapeutic agents, along with the two principal transformation pathways used to modulate TAM behavior. Morrissey et al. developed first‐generation CAR‐M therapy, demonstrating that engineered macrophages with CAR‐based phagocytic modules could engulf both tumor‐associated antigens and whole cancer cells. The incorporation of intracellular domains from multiple EGF‐like domains 10 (Megf10) and FcRγ greatly increased cancer cell phagocytosis [133]. In 2019, an anti‐HER2–CD147 CAR‐M construct was developed to target HER2‐expressing tumor cells. Engagement of HER2 activated CD147 signaling, which induced MMP secretion and degraded tumor stroma, thereby enhancing T‐cell infiltration into the TME [134].

Second‐generation CAR‐M development has focused on optimizing structural design to enhance antigen‐presenting capacity and prolong maintenance of the M1 phenotype. CAR‐M platforms must also enable efficient expansion to clinically relevant quantities and support derivation from multiple cellular sources [45]. Zhang et al. introduced first‐ and second‐generation CAR‐iMacs. iPSCs were generated from peripheral blood mononuclear cells (PBMCs) of healthy donors using a nonintegrating, episomal vector that encoded reprogramming factors; this was followed by the isolation of individual iPSC clones. Conventional CAR constructs were introduced into iPSCs via lentiviral transduction. CAR‐iMacs expanded more than 50‐fold compared with PBMC‐derived macrophages and remained viable for more than 30 days. CAR transgene expression reached approximately 85%, and CD11b/CD14 expression approached 100%. In an ovarian cancer mouse model, CAR‐iMac treatment combined with IFN‐γ administration reduced tumor burden relative to controls [135]. However, first‐generation CAR‐Ms were likely to undergo conversion into an M2‐like, tumor‐promoting phenotype in the immunosuppressive TME. To overcome this limitation, Zhang et al. developed a second‐generation CAR‐M approach capable of sustaining M1 polarization. They incorporated the intracellular Toll/IL‐1 receptor (TIR) signaling domain of TLR4—known to promote macrophage activation and M1 polarization—into the intracellular region of the CAR construct. TIR‐CAR‐iMacs generated by iPSC differentiation and gene editing exhibited enhanced M1 polarization stability. A second‐generation CAR‐M design was established by linking CD3ζ with the TIR domain. This construct facilitated considerably greater targeted antitumor activity and promoted the secretion of IL‐6, IL‐12, IL‐23, TNF, and other proinflammatory cytokines; it also demonstrated strong antigen‐presentation capacity. The constructed maintained a CD80 positivity rate above 80% and sustained an M1 phenotype for more than 7 days, enabling resistance to the immunosuppressive TME [136]. Building on this platform, Zhang et al. reported in 2023 that knockout of the aconitate decarboxylase 1 (*ACOD1*) gene enhanced CAR‐iMac polarization toward a proinflammatory state. This modification increased reactive oxygen species production, improved phagocytosis, and strengthened cytotoxic activity against cancer cells in vitro. *ACOD1*‐deficient CAR‐iMacs demonstrated superior tumor suppression and improved survival in mouse models [137]. THP‐1 cells can also be driven toward an M1 phenotype by LPS and IFN‐γ. In 2020, Klichinsky et al. used an adenoviral vector (Ad5f35) to generate anti‐HER2–CD3ζ CAR‐Ms from human THP‐1 macrophages. These CAR‐Ms repolarized M2 macrophages into an M1 phenotype and maintained this state for at least 40 days, leading to sustained proinflammatory cytokine secretion, alleviation of the immunosuppressive TME, and enhanced survival in tumor‐bearing mouse models [132].

Third‐generation CAR‐M designs focus on achieving macrophage reprogramming via stable, scalable nanotechnology and in vivo induction. In 2021, Kang et al. utilized the nanocarrier mannose‐based polyethyleneimine to deliver a *piggyBac* transposon plasmid encoding CAR–IFN‐γ into macrophages, inducing CAR‐M formation in situ. Among transfected cells, 82% of CAR‐expressing cells were macrophages and 6% were dendritic cells. IFN‐γ promoted repolarization of M2 macrophages into the M1 phenotype, thereby enhancing CAR‐M–mediated tumor phagocytosis and strengthening tumor‐specific cytotoxic T lymphocyte responses [138]. In 2023, Gao et al. designed a synthetic DNA nanocarrier composed of a polyglutamic acid shell conjugated to RP‐182, a macrophage‐targeting peptide. The carrier encapsulated a *piggyBac* transposon plasmid expressing an erythroblastic leukemia viral oncogene homolog 2 (ErbB2) single‐chain variable fragment–CAR, enabling efficient macrophage‐specific CAR integration and activation [139]. Also in 2023, researchers developed nonviral nanocarriers to directly generate CAR‐Ms in vivo for solid tumor treatment. CD24 functions as a major innate immune checkpoint in cancer by engaging the inhibitory receptor Siglec‐10 on TAMs, promoting immune evasion [140]. To counter this mechanism, two mRNAs—encoding a CAR and a truncated Siglec‐G receptor variant (lacking the immunoreceptor tyrosine‐based inhibitory motif; i.e., Siglec‐GΔITIM)—were packaged into lipid nanoparticles targeting liver macrophages. Siglec‐GΔITIM relieved CD24‐induced immunosuppression, significantly enhanced TAM phagocytic activity in HCC models, and inhibited tumor progression [141].

In 2024, a preprint reported evaluation of 36 distinct immunogenic cell death signals delivered via lipid nanoparticle–mediated mRNA targeting of macrophages. These signals included phagocytosis‐promoting constructs such as CD3ζ and Dectin‐1, as well as the proinflammatory receptors CD40 and TLR4. In particular, CAR‐Ms programmed with CD3ζ or TLR4 strongly promoted in vivo repolarization of M2 macrophages into the M1 phenotype, while modulating NF‐κB signaling to increase PD‐L1 and MHC‐I expression levels. This enhancement improved T‐cell activation and increased tumor‐cell susceptibility to T cell–mediated cytotoxicity, thereby suppressing tumor progression. CAR‐M therapy also remodeled the immunosuppressive TME, increasing the abundance of T‐cell factor 1 (TCF1)+ programmed cell death‐1 (PD‐1)+ progenitor‐exhausted CD8+ T cells. Furthermore, the combination of PD‐L1 blockade with CAR‐M therapy amplified TAM antigen presentation and strengthened antitumor immune responses.

#### 4.2.2 Current Status of CAR‐M–Related Clinical Research

To date, six CAR‐M–related clinical trials have been initiated [142, 143]. In 2018, MaxCyte developed MCY‐M11 (NCT03608618), an autologous cell therapy based on mRNA‐transfected PBMCs expressing a single‐chain variable fragment targeting human mesothelin (MSLN) as the antigen‐binding domain [144]. This product is intended for patients with MSLN‐expressing ovarian cancer and peritoneal mesothelioma. In 2020, TAK‐102 (NCT04405778) entered clinical evaluation for the treatment of GPC3‐positive solid tumors. In 2021, anti‐HER2 CAR‐M therapies were tested in relapsed or refractory HER2‐positive solid tumors and breast cancer (NCT04660929 and NCT05007379). In NCT04660929, Carisma Therapeutics evaluated CT‐0508, an adenovirus‐transduced CAR‐M product targeting HER2‐overexpressing tumors. This Phase 1 study enrolled 18 patients and represented the first clinical assessment of adenoviral CAR‐M engineering. On March 20, 2021, Carisma Therapeutics announced completion of the Phase 1 trial of CT‐0508. NCT05007379 is another Phase 1 trial examining anti‐HER2 CAR‐M therapy in patients with HER2‐positive breast cancer. In 2022, TAK‐103 (NCT05164666), another MSLN‐targeted CAR‐M candidate, entered clinical evaluation for advanced or metastatic solid tumors expressing MSLN. In November 2023, the US Food and Drug Administration approved CT‐0525 (NCT06254807) for Phase 1 clinical testing in patients with HER2‐positive locally advanced or metastatic solid tumors. Another ongoing Phase 2 study, NCT06224738, is investigating bone marrow stem cell–derived HER2‐targeted CAR‐M therapy for advanced HER2‐positive gastric cancer (Table 1).

**TABLE 1:** CAR‐M–related clinical trials.

Clinical trials identifier	CAR‐M product	Target	Cell source	Conveying method	Generation method	Disease	Phase
**NCT03608618**	MCY‐M11	MSLN	PBMC	Intraperitoneal reinfusion	mRNA	Ovarian cancer and peritoneal mesothelioma	Phase 1
**NCT04405778**	TAK‐102	GPC3	Autologous white blood cells	Intravenous reinfusion	N/A	GPC3‐positive solid tumors	Phase 1
**NCT04660929**	CT‐0508	HER‐2	PBMC	Intravenous infusion	Adenoviral vector system	HER2 overexpressing solid tumors	Phase 1
**NCT05007379**	HER2 CAR‐M	HER‐2	N/A	N/A	N/A	Breast cancer	Phase 1
**NCT05164666**	TAK‐103	MSLN	Autologous white blood cells	Intravenous infusion	N/A	Advanced or metastatic solid tumors	Phase 1
**NCT06254807**	CT‐0525	HER‐2	Monocyte	Intravenous reinfusion	N/A	HER2 overexpressing solid tumors	Phase 1
**NCT06224738**	MAC‐001	HER‐2	Bone marrow stem cells	Intraperitoneal reinfusion	Adenoviral vector system	HER2‐positive advanced gastric cancer	Early Phase 1
**NCT05138458**	MT‐101	CD5	Myeloid cell	Intravenous reinfusion	mRNA	CD5‐positive peripheral T‐cell lymphoma	Ongoing Phase 1/2

*Data Source*: ClinicalTrials.gov.

Despite continued clinical progress, the development of CAR‐M therapy faces substantial challenges. First, the immunosuppressive nature of the TME remains a major barrier to efficacy. Persistent immunosuppressive cytokines, metabolites, and metabolic constraints can greatly diminish CAR‐M antitumor activity [145]. Second, CAR‐M durability and phenotypic stability are limited; it is difficult to maintain a proinflammatory M1 state in vivo, and CAR‐Ms may gradually repolarize toward an M2 phenotype within the TME, reducing therapeutic effectiveness [146, 147]. Delivery and manufacturing limitations also present bottlenecks. Although viral vectors provide high transduction efficiency, they carry safety concerns. Nonviral delivery systems, such as mRNA and nanomaterial‐based platforms, offer advantages in immunogenicity and controllability but require further optimization to improve efficiency and stability [148, 149]. Additionally, potential adverse reactions—including excessive inflammatory cytokine release and off‐target effects—have not yet been fully assessed in early‐phase trials [150]. To address these limitations, several improvement strategies have been proposed. These include optimization of CAR structures (e.g., integration of TLR or CD40 signaling domains) to promote sustained M1 polarization and enhance antitumor activity [151]; development of advanced nanodelivery platforms and in vivo programming methods to improve delivery efficiency and safety [152]; and exploration of combination immunotherapies—such as pairing CAR‐M with immune checkpoint inhibitors—to remodel the TME and increase clinical efficacy [153, 154]. Although CAR‐M therapy remains in the early stages of clinical development, continued progress in CAR design, delivery technologies, and combination strategies supports its promising potential for the treatment of solid tumors.

### 4.3 Macrophage Delivery Systems and Exosome Therapy

#### 4.3.1 Engineered Macrophages as Anticancer Drug Carriers

Similar to nontargeted erythrocytes and neutrophils, M1 macrophages—such as those derived from bone marrow or RAW264.7 cells—can serve as efficient carriers for drug delivery and offer unique advantages in cancer therapy. M1 macrophages can phagocytose foreign particles and circulate throughout the body via the bloodstream. Additionally, their surface α4 and β1 integrins interact with vascular cell adhesion molecule‐1 on cancer cells, enabling specific binding to tumor tissues [130]. Currently, three main strategies exist for incorporating macrophages into drug delivery:

1. Using macrophages directly as drug carriers. Fu et al. demonstrated that loading doxorubicin into RAW264.7 macrophages through simple incubation produced favorable anticancer effects, including tumor suppression, prolonged survival, and reduced metastasis in 4T1 tumor‐bearing mice [155]. Huang et al. developed engineered macrophages (Oxa(IV)@ZnPc@M) carrying nanomedicine that contains an oxaliplatin prodrug and a photosensitizer. This near‐infrared–activated platform effectively eliminated primary tumors through combined chemotherapy and photodynamic therapy while inducing immunogenic cell death [156]. However, certain anticancer drugs used to deplete TAMs in the TME may exert cytotoxic effects on M1 macrophage carriers. For instance, cisplatin and gemcitabine inhibit M1 macrophage proliferation [157]. Therefore, when utilizing macrophages as direct drug carriers, potential cytotoxic effects on macrophage viability and function must be carefully considered.
2. Using macrophages indirectly as drug carriers. The incubation of anticancer drug–containing liposomes or nanoparticles with live macrophages enables generation of liposome‐ or nanoparticle/drug–loaded macrophages [158]. This approach both reduces drug‐induced cytotoxicity toward macrophages and enhances drug‐loading efficiency. Additionally, macrophages loaded with liposomes or nanoparticles generally show superior tumor therapeutic effects relative to directly drug‐loaded macrophages or liposomes/nanoparticles alone. Li et al. demonstrated that macrophages loaded with SOC (N‐succinyl‐N′‐octyl chitosan)–paclitaxel (PTX) particles produced stronger therapeutic effects than macrophages directly carrying small‐molecule agents such as doxorubicin or PTX; SOC‐PTX–loaded macrophages reduced tumor volume by approximately 93%, compared with reductions of ∼66% and ∼69% for doxorubicin‐ and PTX‐loaded macrophages, respectively [159]. Choi et al. similarly reported that mouse peritoneal macrophages loaded with liposomal doxorubicin (macrophage–LP‐Dox) inhibited tumor growth more effectively than LP‐Dox alone [160]. Tao et al. synthesized PTX‐loaded nanoformulations (nano‐PTX) and observed that bone marrow–derived macrophage–nano‐PTX complexes more efficiently inhibited U87 glioma cell growth compared with nano‐PTX alone [161]. Ikehara et al. treated tumors via intraperitoneal macrophages loaded with oligomannose‐coated liposomes containing 5‐fluorouracil. Notably, oligomannose‐coated liposome–encapsulated 5‐fluorouracil alone did not significantly inhibit tumor growth [162]. Nanoparticle/drug–loaded M1 macrophages can also cross the blood–brain barrier. Pang et al. generated M1 macrophage–loaded nanoparticles by incubating PLGA nanoparticles with primary M1 macrophages. In vivo imaging demonstrated that macrophage–loaded nanoparticles accumulated in brain tumors more effectively compared with free nanoparticles. Furthermore, nanoparticles carrying both doxorubicin and macrophages significantly enhanced antiglioma activity, prolonged median survival, and increased tumor cell apoptosis [163]. However, this indirect delivery strategy has limitations. Nanoparticle drugs internalized via macrophage endocytosis become sequestered in phagosomes, which may reduce drug release efficiency. Additionally, macrophages may degrade the loaded nanoparticles, and the nanoparticles themselves may impair macrophage viability [164, 165]. To address these issues, Doshi et al. developed “phagocytosis‐resistant backpacks,” in which nanoparticle drugs were engineered to adhere to the macrophage surface without undergoing internalization or disrupting macrophage tumor‐targeting capabilities [164]. Holden et al. also modified macrophage surfaces to create hybrid macrophage–nanoparticle systems. Nanoparticles (QD525 or AAFG4.5‐PEG) and polyamidoamine dendrimers were immobilized on RAW264.7 macrophage surfaces via transient Schiff base linkages or more stable amine linkages [166].
3. Using macrophage membrane–coated nanoparticles as drug carriers. Several membrane proteins on M1 macrophages exhibit strong tumor‐targeting and deep‐penetration properties. Thus, the use of isolated M1 macrophage membranes to coat nanomedicines can enhance targeted accumulation of therapeutic agents within tumors. Li et al. developed macrophage membrane–coated nanogemcitabine particles designed to promote lymphocyte infiltration and synergize with anti–PD‐L1 therapy to restore exhausted lymphocytes. These particles demonstrated effective intratumoral penetration, responsive drug release, and enhanced lymphocyte infiltration into tumor regions [167]. Xuan et al. generated macrophage membrane–camouflaged mesoporous silica nanocapsules for doxorubicin delivery in 4T1 tumor models. Their engineered nanocapsules exhibited prolonged blood circulation and significantly greater tumor accumulation than unmodified nanoparticles, resulting in complete tumor eradication [168]. Approaches that harness the natural tumor‐targeting properties of M1 macrophages and engineer them as drug delivery vehicles enable precise delivery of therapeutic agents while bypassing immune barriers within the TME. This strategy also reduces potential toxicity to healthy tissues. Although not yet implemented in clinical settings, continued optimization and refinement may establish M1 macrophage–based delivery systems as promising platforms for precision‐targeted cancer therapy.

#### 4.3.2 Macrophage‐Derived Exosome–Targeted Therapy

Exosomes are small membranous vesicles (30–150 nm) that contain proteins, growth factors, cytokines, lipids, microRNAs, mRNAs, and DNA. They originate from the inward budding of endosomal membranes and are released into the extracellular space when multivesicular bodies fuse with the plasma membrane. Exosomes are secreted by multiple cell types, including macrophages, reticulocytes, dendritic cells, B lymphocytes, and tumor cells. They modulate signal transduction pathways and function as intermediaries of intercellular communication [168]. Macrophage‐derived exosomes retain membrane features similar to their parent cells, enabling them to serve as effective carriers for anticancer therapeutics. Kim et al. incorporated PTX into macrophage‐derived exosomes via sonication. PTX‐loaded exosomes exhibited more than a 50‐fold increase in cytotoxicity in drug‐resistant MDCK_MDR1_ (Pgp+) cells and produced a potent anticancer effect in a Lewis lung carcinoma mouse model [169]. To address barriers posed by the blood–brain barrier and tumor hypoxia in glioblastoma, Wu et al. encapsulated catalase into silica nanoparticles to alleviate hypoxia. These nanoparticles were then coated with AS1411 aptamer–modified macrophage exosomes, which demonstrated efficient blood–brain barrier penetration and strong tumor‐targeting properties. After tumor cell endocytosis, the freed catalase decomposed hydrogen peroxide (H_2_O_2_) to generate molecular oxygen (O_2_), thereby relieving hypoxia [142]. Additionally, recent studies have shown that TAMs secrete large numbers of PD‐L1–enriched exosomes, which strongly suppress CD8+ T‐cell proliferation and function [143, 170, 171, 172]. Exosome secretion by TAMs is driven by mitogen‐activated protein kinase–activating death domain protein (MADD)‐mediated guanosine triphosphate loading of Rab27a, a key regulator of exosome release. Protein kinase B–dependent phosphorylation of MADD at serine 70 initiates this process. Zhong et al. used C12‐200 lipid nanoparticles delivering *RAB27a* siRNA in combination with anti–PD‐1 therapy in the YUMM1.7 melanoma model, which is resistant to PD‐1 blockade. *RAB27a* silencing in TAMs reduced the release of immunosuppressive exosomes, enhanced T‐cell activation, and sensitized tumors to anti–PD‐1 treatment [19]. Therefore, targeting the secretion or function of TAM‐derived exosomes represents a promising strategy to enhance tumor immunotherapy (Table 2).

**TABLE 2:** Macrophage‐related cancer therapies.

Related agents	Mechanism of action	Functional effect	Strategy of TAM regulation	References
Microtubule inhibitors (e.g., Paclitaxel)	Activates NF‐κB signaling pathway, directly promotes macrophage polarization toward M1 phenotype	Enhances clearance of triple‐negative breast cancer cells	Reprograms TAMs to M1‐like phenotype, converts immunosuppressive M2 TAMs to antitumor M1 TAMs	[113]
Vincristine	Stabilizes microtubules, induces TAMs to polarize into M1 subtype	Enhances phagocytic ability of macrophages		[114]
DCP1 (sugar analog)	Upregulates genes associated with M1 polarization and phagocytosis	Drives M2‐like macrophages to express M1 signature genes		[116]
NR_109 knockdown	Inhibits c‐Myc transcription	Inhibits TAM polarization to M2‐like phenotype		[117]
CSF‐1/CSF‐1R signaling pathway inhibitors	Reprograms TAMs to M1 phenotype	Converts M2 TAMs into antitumor M1 phenotype		[118, 119, 120]
CSF‐1/CSF‐1R and PD‐L1 immune checkpoint inhibitors (combination therapy)	Regulates TAM polarization and activates CD8+ T cells	Promotes M2‐to‐M1 repolarization of TAMs and activates CD8+ T cell response		[122]
PI3K‐γ inhibitor (e.g., NVP‐BEZ235)	Reprograms TAMs polarization	Converts immunosuppressive TAMs to antitumor M1 phenotype		[121]
Immunomodulator α‐1 (Tα‐1) combined with Sofosbuvir	Activates TLR7/MyD88–SHIP1 signaling, inhibits TBK1 phosphorylation and IL‐10 expression	Inhibits M2 polarization of TAMs and tumor cell proliferation in vivo		[123]
TLR7/8 agonist (e.g., Resiquimod, R848)	Induces M2‐to‐M1 repolarization of TAMs	Improves immune suppression in tumor microenvironment (TME)		[124, 125]
Anti‐CD5L monoclonal antibody	Regulates TAM polarization	Improves immunosuppressive status of TME		[129]
Nanoparticle‐encapsulated Paclitaxel	Promotes M1 macrophage‐mediated phagocytosis	Enhances tumor infiltration and antitumor immunity	Engineered macrophages as antitumor agents	[126, 127, 128]
RAB27a siRNA + PD‐1 antibody	Inhibits exosome secretion and tumor‐promoting signals	Enhances TAM activity, increases PD‐1 therapy sensitivity	Targets TAMs to inhibit exosomal immunosuppression	[19]

## 5 TAMs as Diagnostic, Prognostic, and Predictive Biomarkers

As core regulators of the TME, TAMs have emerged not only as therapeutic targets but also as potential biomarkers for diagnosis, prognosis, and prediction of treatment response. This section outlines how detection techniques and multiomics analyses have advanced the clinical utility of TAMs across these domains. First, we summarize current approaches for assessing TAMs, ranging from conventional immunohistochemistry (IHC) and flow cytometry to advanced single‐cell and spatial transcriptomic platforms. Collectively, these technologies delineate the phenotypic and spatial heterogeneity of TAM populations. Next, we examine the prognostic relevance of TAM density, polarization state, and spatial distribution across multiple tumor types, emphasizing their strong associations with clinical outcomes. Finally, we highlight recent progress in identifying TAM‐related biomarkers that predict therapeutic response, including metabolic signatures and critical signaling pathways. Overall, these findings underscore the value of TAMs as comprehensive indicators that integrate molecular pathology with precision oncology.

### 5.1 Evaluation Methods

TAMs are key immune cells in the TME. Their evaluation methods have progressed from conventional histological staining to high‐dimensional, multiomics technologies, enabling systematic analysis from macroscopic tissue distribution to single‐cell and spatial resolutions [173]. Conventional IHC and immunofluorescence methods are widely used to detect TAM markers and assess spatial localization because they provide intuitive visualization of tissue sections and are technically accessible. Commonly used markers include CD68, CD163, and CD206, with the CD163/CD68 ratio frequently applied to assess immunosuppression and prognosis [174, 175]. The development of multiplex IHC/immunofluorescence methods has increased analytical throughput by enabling simultaneous detection of more than 10 protein markers, thus enhancing the accuracy of TAM subset characterization and spatial mapping [176]. However, these techniques cannot fully reflect functional states at the transcriptomic level. Flow cytometry and mass cytometry (e.g., cytometry by time‐of‐flight) have been adopted to profile complex phenotypes and cytokine expression at the single‐cell level, distinguishing proinflammatory from immunosuppressive TAM subsets [173, 177]. Although these approaches support high‐parameter analysis, spatial information is lost during tissue dissociation, limiting evaluation of TAM interactions within the tissue microenvironment. scRNA‐seq has advanced understanding of TAM molecular heterogeneity by identifying new subsets beyond the classical M1/M2 framework and by reconstructing differentiation trajectories that reveal TAM plasticity in immune regulation and tumor progression [178, 179, 180]. However, scRNA‐seq also lacks spatial context. Spatial transcriptomics addresses this limitation by integrating tissue imaging with transcriptomic profiling to reveal the spatial organization of TAMs and their functional roles within distinct tumor regions [181, 182]. Spatial transcriptomics studies show that TAMs in different tumor compartments exhibit distinct functional tendencies—for example, immunosuppressive phenotypes predominate in tumor cores, whereas edge‐ or vessel‐associated TAMs tend to promote angiogenesis or regulate T‐cell function [183, 184]. Spatial transcriptomics has also demonstrated complex spatial interaction networks between TAMs and other immune cells, including Tregs [181, 185]. These spatial characteristics are closely linked to variations in immunotherapy response. Current research increasingly incorporates multimodal integration strategies—such as combining scRNA‐seq, spatial transcriptomics, and multiplex IHC—to achieve comprehensive evaluation of TAM phenotypes, transcriptional states, and spatial organization. These approaches also enhance analysis of TAM interactions with other immune cells, supporting therapeutic target discovery and precision treatment development [186]. Overall, TAM evaluation is shifting from low‐dimensional morphological and phenotypic characterization to high‐dimensional, multiomics, spatially resolved analysis, establishing a foundational framework for clinical classification, prognostic assessment, and prediction of treatment response.

### 5.2 Prognostic Value

TAMs are key immune regulatory cells in the TME. Their density, polarization state (such as M1 or M2), and spatial distribution across tumor regions are closely associated with clinical prognosis in multiple cancer types [187]. High densities of M2 TAMs commonly accelerate tumor progression through mechanisms that include immunosuppression, angiogenesis, and epithelial–mesenchymal transition; thus, they are associated with poor outcomes. In contrast, M1 TAMs tend to exert antitumor immune activity and display links to more favorable survival indicators [188, 189]. In breast cancer, CD163+ M2 TAMs are widely recognized as central drivers of the immunosuppressive microenvironment. They activate the CSF1/CSF1R and IL‐10/STAT3 pathways, induce T‐cell exhaustion and metastatic phenotype switching, promote tumor infiltration and metastasis, and substantially shorten disease‐free and overall survival [190, 191]. In contrast, M1 TAMs upregulate IL‐12 expression through NF‐κB/RelA‐mediated signaling, thereby activating Th1 immunity and strengthening CD8+ cytotoxic T‐cell function. Enrichment of M1 TAMs is consistently associated with increased immune infiltration, reduced recurrence risk, and prolonged survival [192].

In HCC, TAMs play a critical role in sustaining an immunosuppressive microenvironment and facilitating tumor progression via STAT3 activation. Li et al. demonstrated that M2 TAMs regulate immune‐related gene expression through a STAT3‐dependent mechanism, enhance tumor cell proliferation and migration, and strongly promote invasion [193]. TAMs also secrete VEGF, TGF‐β, and MMP9, which induce angiogenesis and epithelial–mesenchymal transition, further increasing metastatic potential and elevating the risk of postoperative recurrence [194]. Additional studies have shown that the FOLR2+ TAM subpopulation suppresses antitumor immune responses in HCC by recruiting or activating Tregs. The abundance of these FOLR2+ TAMs is strongly associated with worse clinical outcomes [195].

In non–small cell lung cancer (NSCLC), TAMs play a central role in shaping the immunosuppressive TME. Studies have shown that M2 TAMs express high levels of the immunosuppressive cytokine IL‐10 and the chemokine CCL22, which recruit Tregs through activation of the JAK1/STAT1/NF‐κB pathway. Such recruitment inhibits the effector function of CD8+ cytotoxic T lymphocytes and promotes immune escape [196, 197]. This mechanism weakens antitumor immunity and is strongly associated with resistance to immune checkpoint inhibitor therapy, resulting in reduced treatment response and shortened overall survival [198]. Clinical cohort studies have also demonstrated that increased total TAM density in NSCLC tissues and elevated ratios of the M2 marker CD163 to the pan‐macrophage marker CD68 (i.e., CD163/CD68 ratio) are associated with advanced tumor stage, higher metastatic risk, and poor prognosis. These indicators have been proposed as independent prognostic biomarkers for patient risk stratification [199].

In ovarian cancer, TAMs are predominantly polarized to the M2 phenotype. These cells contribute to matrix remodeling, angiogenesis, and extracellular matrix reconstruction by secreting IL‐6, IL‐10, TGF‐β, and matrix metalloproteinases, thereby supporting tumor cell migration, peritoneal implantation, and metastasis [200, 201]. In colorectal cancer, enrichment of CD163+ TAMs is associated with higher tumor grade, increased expression of epithelial–mesenchymal transition markers, and T‐cell exhaustion. TAMs form a positive feedback loop by secreting TGF‐β and MMP9, further promoting epithelial–mesenchymal transition and driving circulating tumor cells into a highly invasive state that can traverse the vascular wall and establish distant metastases [202]. Therefore, TAM density, phenotype, and functional status serve as important biomarkers for assessing metastatic potential and predicting disease‐free survival.

In addition to highly prevalent solid tumors such as breast cancer and HCC, M2 TAMs are also commonly observed in oral squamous cell carcinoma, pancreatic ductal adenocarcinoma, bladder cancer, and other malignancies, where they are strongly associated with immunosuppression, epithelial–mesenchymal transition, and metastatic progression. In oral squamous cell carcinoma, CAF‐secreted aortic carboxypeptidase‐like protein chemotactically recruits M2 TAMs and Tregs, thereby establishing a highly immunosuppressive microenvironment. This environment strongly inhibits CD8+ T‐cell infiltration and activity, induces epithelial–mesenchymal transition and stemness programs in cancer cells, and promotes enhanced tumor invasiveness and immune escape [203]. In pancreatic ductal adenocarcinoma, TAMs are central drivers of immune tolerance. M2 TAMs activate the CSF1/CSF1R axis in response to signals such as heat shock protein (HSP)90α secreted by CAFs and tumor cells. This activation recruits Tregs, upregulates PD‐L1 expression, inhibits CD8+ effector T‐cell function, and establishes a profoundly immunosuppressive “cold” TME that is resistant to immune checkpoint inhibitors [204, 205]. Concurrently, TAMs cooperate with CAFs to remodel the extracellular matrix, secrete TGF‐β and MMP9 to promote epithelial–mesenchymal transition and angiogenesis, and enhance both local invasion and distant metastasis [206]. A similar high degree of TAM–CAF–extracellular matrix synergy has been described in bladder cancer and other solid tumors. This “immune–stroma” axis forms a complex ligand–receptor interaction network that regulates immune checkpoint expression, activates Tregs, sustains tumor stemness, and increases tumor cell motility. It has emerged as a key mechanism driving immune escape, chemotherapy resistance, and metastatic dissemination [206].

As a highly plastic immune cell population, TAMs—particularly their abundance, polarization state, and spatial distribution—have become important biomarkers for prognostic assessment across multiple solid tumors. A deeper understanding of the causal links between TAM biology and tumor progression is expected to support the development of more precise, macrophage‐centered immunotherapeutic strategies.

### 5.3 Predicting Therapeutic Efficacy

TAMs play a dual role in determining immunotherapy response. They can support antitumor immunity or promote immune escape through multiple mechanisms, thereby exerting a strong influence on the efficacy of immune checkpoint inhibitors. In recent years, TAMs have gained recognition as important biomarkers for predicting the therapeutic response to immune checkpoint inhibitors and TAM‐targeted treatments. M2 TAMs usually express high levels of immunosuppressive molecules such as PD‐L1, IL‐10, TGF‐β, and ARG1. They not only directly suppress CD8+ T‐cell activity but also enhance tumor immune tolerance by modulating immune checkpoint pathways, substantially reducing the effectiveness of PD‐1/PD‐L1 blockade. In contrast, M1 TAMs promote antitumor immunity by secreting IL‐12 and activating Th1 responses. Consequently, the M1/M2 ratio has been proposed as a potential biomarker for predicting immunotherapy response [207].

In an HCC model, Liu et al. demonstrated that TAMs are key determinants of immune checkpoint inhibitor efficacy. Enrichment of M2 TAMs was linked to pronounced upregulation of PD‐L1 expression, suppression of T‐cell function, and the development of drug‐resistant phenotypes. Conversely, TAM reprogramming or the inhibition of key pathways such as CSF1/CSF1R significantly enhanced immune checkpoint inhibitor therapeutic response, suggesting that TAM phenotype and immunoregulatory status are both predictors of therapeutic efficacy and potential targets for combinatorial treatment [208]. In solid tumors such as NSCLC and melanoma, M2 TAMs contribute to an “immune‐excluded” phenotype through high expression of PD‐L1, ARG1, TGF‐β, and IL‐10. This environment suppresses CD8+ T‐cell activity and promotes recruitment of Tregs, thereby diminishing the efficacy of immune checkpoint inhibitors [209]. In contrast, M1 TAMs activate the IFN‐γ–dependent STAT1 pathway and upregulate proinflammatory mediators such as MHC‐II and IL‐12, enhancing antigen presentation and cytotoxic T‐cell function. This activity alleviates the immunosuppressive microenvironment and increases responsiveness to immune checkpoint inhibitor therapy [210]. Therefore, the M1/M2 TAM ratio has emerged as a potential biomarker for evaluating therapeutic outcomes in patients receiving PD‐1/PD‐L1 inhibitors.

The predictive value of TAMs in targeted therapy has gained increasing recognition, particularly with respect to CSF1/CSF1R axis inhibition, which has shown considerable therapeutic potential. CSF1R is a critical signaling mediator involved in the survival and maintenance of the M2 TAM phenotype. It is highly expressed across multiple tumor types and has become an important therapeutic target for immunosuppressive TAMs. Studies have shown that blockade of the CSF1/CSF1R pathway reduces the number of M2 TAMs and decreases PD‐L1 and ARG1 expression; it also reshapes the tumor immune microenvironment and enhances sensitivity to immune checkpoint inhibitor therapy [211, 212]. Additionally, the CCL2/CCR2 axis plays a central chemotactic role in TAM recruitment. In breast cancer, pancreatic cancer, and other solid tumors, high tumor CCL2 expression is associated with improved responsiveness to CCR2 or CSF1R inhibitors, indicating that CCL2 expression levels may serve as a predictive biomarker concerning the efficacy of TAM‐targeted therapy [213]. Notably, combination therapy with CSF1R inhibitors and PD‐L1 antibodies has demonstrated synergistic effects in multiple preclinical models and early clinical studies. This combination substantially enhances antitumor immune responses by simultaneously reducing the proportion of immunosuppressive TAMs and alleviating T‐cell functional suppression [214, 215].

At the metabolic level, the energy metabolism patterns of TAMs not only determine their polarization state and functional activity but also directly influence their ability to regulate immunotherapy responses and their performance in cell‐based treatments. Previous studies have shown that M2 TAMs primarily rely on OXPHOS and fatty acid oxidation. This stable and efficient metabolic profile supports maintenance of the immunosuppressive phenotype, sustained secretion of inhibitory cytokines such as TGF‐β and IL‐10, and promotion of tumor angiogenesis and immune escape [216, 217]. In contrast, M1 TAMs use glycolysis as their dominant energy source. They exhibit high metabolic activity, rapidly respond to pathological stimuli, secrete proinflammatory cytokines such as IL‐12 and TNF‐α, and enhance antigen presentation, thereby activating Th1 and CD8+ cytotoxic T cells to exert potent antitumor effects [218]. This metabolic plasticity regulates TAM immune function and defines their predictive value in emerging immunotherapies. In CAR‐M therapy, constructs tend to maintain a glycolysis‐dominant M1 metabolic program, enhancing their survival, migration, and antigen‐presentation capacity within the TME [219]. CAR‐M populations with high glycolytic flux demonstrate stronger tumor‐suppressive effects in murine transplantation models, whereas OXPHOS‐biased TAMs fail to sustain comparable antitumor activity [220]. Thus, the OXPHOS/glycolysis metabolic ratio provides a potential metabolic marker for evaluating TAM immune function and predicting the therapeutic efficacy of CAR‐Ms and other TAM‐targeted approaches.

The predictive value of TAM‐associated molecules and their signaling pathways is becoming increasingly prominent in immunotherapy and macrophage‐centered therapeutic strategies. Multidimensional indicators—including PD‐L1, ARG1, CSF1R, the CCL2/CCR2 chemotactic axis, the M1/M2 polarization ratio, and metabolic states defined by glycolysis versus OXPHOS—are being incorporated into emerging multiparameter prediction models centered on TAM biology. Through rapid advances in high‐dimensional omics technologies such as single‐cell transcriptomics, spatial transcriptomics, and mass spectrometry, more refined delineation of TAM subpopulations, functional states, and dynamic interaction networks will become possible. This progress will improve the precision of pretreatment risk stratification and therapeutic response prediction, providing a strong theoretical and technical foundation to optimize TAM‐targeted combination immunotherapy strategies. Ultimately, TAMs may transition from being considered “prognostic markers” to serving as “functional decision‐making nodes” in precision oncology.

## 6 Conclusions and Outlook

TAMs exhibit heterogeneity and plasticity within the complex TME. Due to their dual functional roles, they have become a central focus in the development of macrophage‐based cancer therapies. These therapeutic strategies can be broadly grouped into four major approaches. First, regulating macrophage repolarization can suppress the tumor‐promoting effects of M2 TAMs—such as enhanced migration and angiogenesis—while simultaneously leveraging proinflammatory cytokines secreted by M1 TAMs to improve T‐cell infiltration and inhibit tumor progression. Second, direct elimination of M2 TAMs is an effective strategy. Targeting specific TAM receptors, including CSF1R and CD47, can reduce TAM abundance or inhibit suppressive activity. Anti–CSF1R monoclonal antibodies have shown therapeutic benefit in multiple tumor types during clinical trials; combination treatment with chemotherapy can further augment their efficacy. Additionally, blockade of the CD47–signal regulatory protein (SIRP)α axis is an emerging approach that enhances macrophage‐mediated phagocytosis and promotes tumor cell clearance. Third, CAR‐M therapy has rapidly advanced through the development of induced macrophages derived from multiple cellular sources. Structural innovations, along with improvements in programming and delivery technologies, have enabled the emergence of third‐generation CAR‐Ms optimized for solid tumor treatment. Several CAR‐M candidates have entered Phase 1 clinical trials. Combination strategies, such as pairing CAR‐Ms with immunogenic cell death–inducing therapies, may further amplify therapeutic benefit. Moreover, nonviral vector–mediated in vivo programming can reduce manufacturing time and cost while mitigating risks associated with viral vectors. Although therapeutic responses vary among tumor sites and safe in situ infusion techniques have not yet been established, clinical data are required to determine appropriate dosing and safety profiles. Finally, the use of macrophages as cellular drug‐delivery vehicles represents another promising therapeutic approach. Their intrinsic tumor‐homing capability enables targeted delivery of chemotherapeutics, nanoparticles, proteins, or nucleic acids to tumor sites. Consequently, macrophage‐based delivery systems hold considerable potential for improving the precision and efficacy of cancer immunotherapy.

Although the four aforementioned strategies have shown great promise in preclinical studies and early clinical exploration, TAM‐targeted therapies continue to face serious challenges. First, the high heterogeneity and plasticity of TAMs drive dynamic and reversible phenotypic transitions across tumor types, disease stages, and treatment contexts. This complexity reduces the universal applicability of single therapeutic strategies; it may also contribute to treatment resistance and tumor recurrence. Second, most current interventions lack sufficient specificity to selectively inhibit tumor‐promoting M2 TAMs while preserving their essential roles in tissue homeostasis, wound repair, and anti‐infective defense. Additionally, TAMs extensively interact with immunosuppressive components such as CAFs, myeloid‐derived suppressor cells, and Tregs, forming a tightly interconnected signaling network that is difficult to reprogram through single‐target drug interventions.

Future development should prioritize multilayered and multidimensional precision strategies. Systematic elucidation of TAM molecular mechanisms remains essential, particularly through spatial transcriptomics, single‐cell multiomics, metabolomics, and epigenetic profiling to map functional landscapes across spatial niches and macrophage subpopulations, thereby enabling identification of highly specific therapeutic targets. In parallel, engineered macrophage therapies continue to advance rapidly. Logic‐gate CAR‐M design, nonviral vector–mediated in vivo reprogramming, and controllable signaling domains integrated with metabolic or inflammatory cues are expected to improve treatment safety, persistence, and functional precision. The incorporation of artificial intelligence–driven multiomics integration into patient stratification may further support personalized therapy and enhance treatment consistency. In terms of therapeutic modalities, methods that combine TAM‐targeted approaches with immune checkpoint inhibitors, chemoradiotherapy, metabolic regulators, or other immune cell therapies are expected to achieve synergistic, multipathway antitumor effects. Through the continued integration of mechanistic insights and clinical investigation, TAM‐targeted therapy is anticipated to transition from experimental research toward precise, controllable, and scalable clinical applications.

## Author Contributions

**Zhimei Liu** and **Shoulong Deng**: writing the original draft preparation. **Yan Li**, **Jingchao Cao**, and **Yefeng Qiu**: writing – review and editing. **Kun Yu**: supervision. All the authors have read and agreed to the published version of the manuscript.

## Funding

This work was supported by the National Natural Science Foundation of China (Grant No. 32072722).

## Ethics Statement

The authors have nothing to report.

## Conflicts of Interest

The authors declare no conflicts of interest.

## Data Availability

Data sharing is not applicable to this article as no new data were created or analyzed in this study.
